# Solubility parameters of enzymatic hydrolysis lignin: a comparative study of inverse gas chromatography and Hansen solubility sphere

**DOI:** 10.1039/d6ra09398a

**Published:** 2026-10-27

**Authors:** Muhammad Ossama, Juho-Pekka Laakso, Robin Luci, Agung Ari Wibowo, Ehsan Asadi, Petri Uusi-Kyyny, Ville Alopaeus

**Affiliations:** a Aalto University, School of Chemical Engineering, Department of Chemical and Metallurgical Engineering, Research Group of Chemical Engineering P.O. Box 16100 FI-00076 Aalto Finland Muhammad.ossama@aalto.fi

## Abstract

Enzymatic hydrolysis lignin (EHL), a major byproduct of second-generation bioethanol production, is a promising renewable feedstock but remains challenging, in part because systematic solvent screening for EHL processing is largely absent from the literature. In this work, we combine inverse gas chromatography (IGC) with gravimetric solubility measurements to establish a practical, experimentally validated framework for solvent selection in EHL processing. Infinite dilution activity coefficients were measured for 31 probe solvents across 408.15–438.15 K. The IGC data were used to determine Hansen solubility parameters (HSPs): *δ*_D_ = 19.15, *δ*_P_ = 13.01, *δ*_H_ = 8.30 and *δ*_t_ = 24.58 MPa^0.5^ while the interaction radius *R*_0_ = 8.98 MPa^0.5^ was fitted using the gravimetric solubility experiments. Surface properties were also evaluated using the Snyder–Karger model. Polar and hydrogen-bonding interactions were identified as the dominant drivers of EHL–solvent affinity. The resulting HSP solubility sphere was validated against gravimetric solubility measurements for 22 solvents, where bio-based solvents such as *γ*-valerolactone was located within the EHL solubility sphere, highlighting their potential as promising bio-based solvent candidates for further evaluation in lignin processing. This work supports the development of an experimental basis for selecting alternative solvents for EHL processing and contributes to more sustainable routes for lignin valorization in lignocellulosic biorefineries.

## Abstract 2

Inverse gas chromatography and solubility measurements establish a quantitative solvent screening framework for enzymatic hydrolysis lignin, identifying bio-based solvents as promising candidates for sustainable lignin processing.

## Graphical abstract

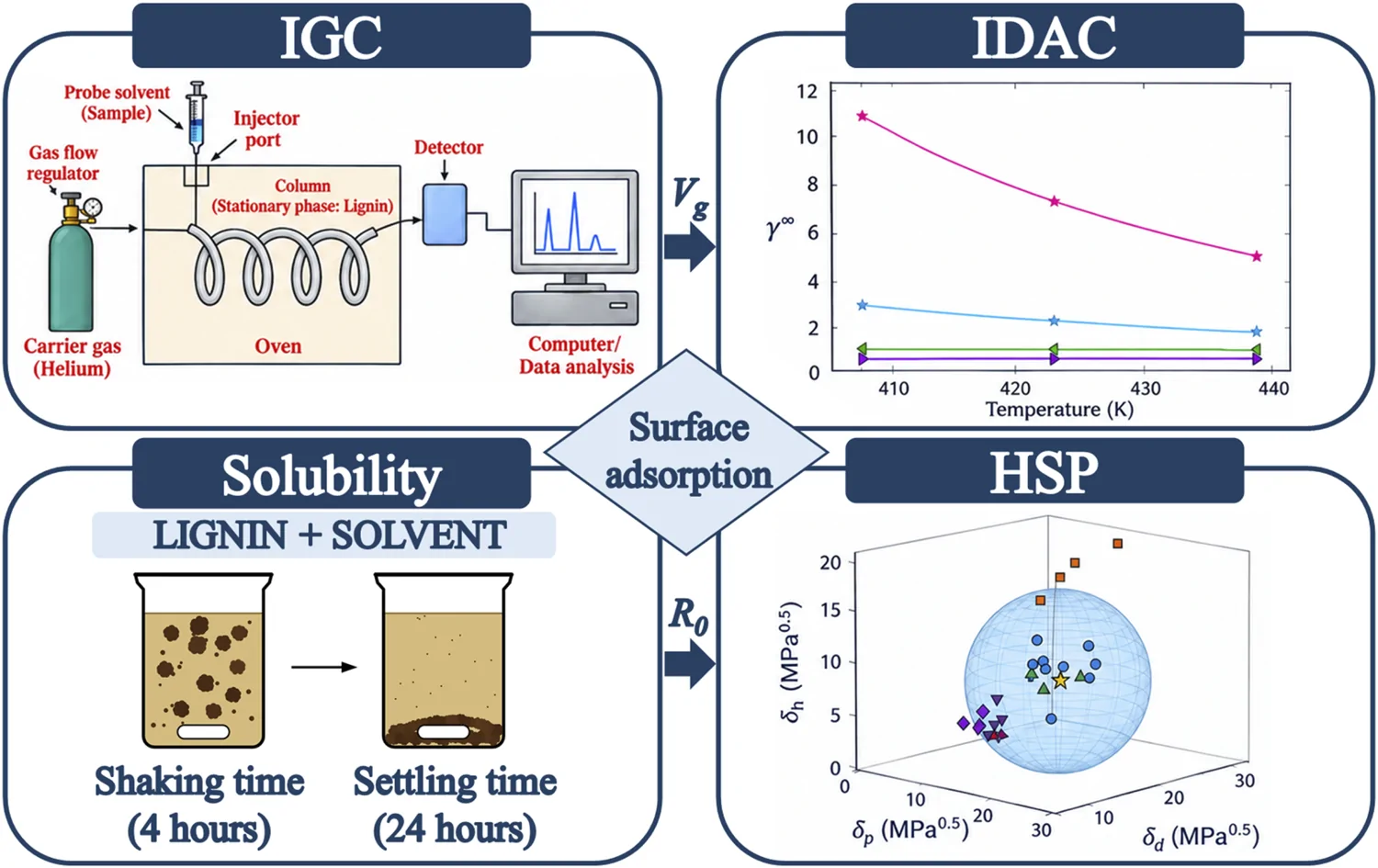

## 1. Introduction

Lignocellulosic biomass is central to the transition away from fossil-derived chemicals and materials. Lignin, a major byproduct of pulping and biorefinery processes, remains substantially underutilized relative to its potential.^1^ Only a small fraction of the 50 million tons of lignin produced across various industrial processes is utilized in the production of value-added products.^2^ Lignin is the second most abundant natural polymer on Earth, and it is composed of three monomeric units: *p*-coumaryl, coniferyl, and sinapyl alcohols.^3^ Due to its heterogeneous structure, high polydispersity index (PDI), and poor solubility in most organic solvents, the valorization of lignin and its derivatives remains a complex task.^1,3^ Enzymatic hydrolysis lignin (EHL), generated *via* processes such as second-generation bioethanol production, is a particularly attractive yet understudied feedstock for value-added chemical and material applications.^4,5^ Furthermore, industrial EHL is influenced by the type of biomass feedstock and the pretreatment processes applied.^6^ Pretreatment methods such as steam explosion and dilute acid treatment can induce lignin condensation, disrupt β-O-4 linkages, and consequently reduce lignin reactivity.^7^ Unlike Kraft or organosolv lignin, EHL is structurally closer to native lignin; combined with its typical sulfur-free nature, high molecular weight, and high phenolic hydroxyl content, this makes it an attractive feedstock for valorization into higher-value products such as lignin bio-oils, phenolic resins, and functional bio-based materials.^8,9^ Despite EHL's industrial relevance, solvent screening, including bio-based solvents, is largely absent from the literature. This presents a critical research gap that this work addresses.

Infinite Dilution Activity Coefficient (*γ*^∞^), quantifies solute–solvent affinity at infinite dilution and is therefore a primary metric for solvent screening. *γ*^∞^ describes the deviation from ideality, where values lower than 1 indicate net attractive (favorable) affinity and values higher than 1 indicate net repulsive (unfavorable) affinity. In lignin, measurements of solvent *γ*^∞^ have been made using Inverse gas chromatography (IGC), since it allows the screening of a large number of solvents.^10,11^

The solubility parameter is widely used to predict compatibility between solute and solvent based on physicochemical parameters such as surface free energy and surface tension.^12^ Hildebrand characterized the solubility parameter as the square root of the cohesive energy density, where chemicals with similar solubility parameters show favorable affinity.^13^ Due to the limitations of Hildebrand solubility parameters for polar solvents, Hansen introduced three-dimensional solubility parameters, or the Hansen solubility parameters (HSPs).^14^ HSPs are based on the assumption that the total cohesive energy density is the sum of contributions from dispersion interaction (*δ*_D_), polar interaction (*δ*_P_), and hydrogen-bonding interaction (*δ*_H_). Within the three-dimensional space, each solvent occupies a fixed point defined by its known HSP value, while the solute is represented by a sphere of interaction radius (*R*_0_) centered at the solute HSP coordinates. The affinity between the solute and the solvent is quantified by the relative energy difference (RED), which is the ratio of solute–solvent distance in Hansen space to the interaction radius. HSP is most widely used as solubility parameters, where solvent screening data are transformed into a fitted compatibility sphere that can be further used to predict the behavior of untested solvents.^15^

IGC has been used in the literature to determine the solubility parameters of polymers such as polymer blends,^16^ ionic liquids,^17^ nanomaterials,^18^ kraft lignin,^19^ and alkali lignin.^10^ Zhao *et al.*^17^ combined IGC with an independently fitted Hansen sphere to characterize ionic liquids, and Liu *et al.*^20^ used HSP to guide lignin fractionation, showing that carbohydrate co-separation depends on a solvent's hydrogen-bonding capacity. Collectively, these studies support the integration of rigorous IGC-derived solubility parameters as a tool for screening solvents in sustainable biomass processing. In IGC, the column is filled with coated material as the stationary phase, where a series of volatile probe solvents are injected at infinite dilution, and the specific retention volume (*V*_g_) is calculated to quantify the affinity of the probe solvent with the stationary phase.^16^

This work aims to investigate the capability of industrial organic solvents commonly used in lignocellulosic biomass conversion and emerging bio-based solvents, including *γ*-valerolactone, dihydrolevoglucosenone, and dimethyl isosorbide. EHL glass transition temperature (*T*_g_), thermal behavior, and molar mass distribution were investigated using differential scanning calorimetry (DSC), thermal gravimetry (TG), and gel permeation chromatography (GPC), respectively. *γ*^∞^, *V*_g_, and temperature-dependent Hildebrand and Hansen solubility parameters for EHL were determined by IGC at three different column temperatures below the glass transition region of EHL. The IGC measurements were also extended above the glass transition region of EHL for three probe solvents. In this work, *R*_o_ was determined from gravimetric solubility experiments due to limited HSP data available for EHL. This EHL-specific HSP dataset will provide a physically grounded foundation for solvent selection throughout EHL processing and also provide a predictive basis for replacing hazardous solvents with renewable alternatives. Beyond EHL, the combined IGC–HSP approach is transferable to other lignin types, thereby supporting bio-based solvent selection for lignin valorization in biorefineries.

## 2. Materials and method

### 2.1. Materials

The EHL was provided by Shandong Long Li Biological Technology Co., Ltd, obtained from corncob conversion to ethanol *via* a microbial enzymatic hydrolysis process. The EHL used in this study was characterized previously at the School of Chemical Engineering, Aalto University.^4,5,21^ The composition of EHL was 91.20 wt% lignin, 7.30 wt% water, 0.12 wt% residual carbohydrate, and 1.42 wt% ash, and the weight percentages of C, O, H, N, and S are 61.29, 29.61, 6.69, 0.98, and 0.01 wt%, respectively. Prior to use, the EHL was dried at 333.15 K in a VWR DRY-line drying oven. The solvents studied in this work include n-alkanes, alcohols, aromatics, esters, ketones, and bio-based solvents (*γ*-valerolactone, dimethyl isosorbide, and dihydrolevoglucosenone). All solvents were purchased from Sigma-Aldrich except ethanol, which was purchased from Anora Oy and dried over 3 Å molecular sieves prior to use. Information on the chemicals studied in this work is provided in Table S1 of the SI.

### 2.2. Determination of molecular weight by gel permeation chromatography (GPC)

The average molecular weight of EHL was determined using an Agilent HPLC system (1100 series, Santa Clara, USA). The calibration curve was obtained with polystyrene sulfonate standards (1000–64 000 g mol^−1^), ascorbic acid (176 g mol^−1^), and NaCl (58 g mol^−1^; detection with refractive index detector). Molar masses were determined using a UV detector at 280 nm, and the columns used were polymer standards service MCS 300 × 8 mm (three columns with pore sizes of 100 Å, 500 Å, and 1000 Å). NaOH was used as eluent at a flow rate of 0.7 ml min^−1^ and a concentration of 0.1 M. The EHL sample was dissolved in the eluent at a concentration of 2 mg ml^−1^.

### 2.3. Thermal analysis

The glass transition temperature of EHL was determined using DSC (TA Instruments Discovery DSC 250). EHL samples (8 ± 1 mg) were hermetically sealed in Tzero aluminum pans, along with an empty, closed Tzero aluminum pan as a reference, and subjected to two heating and cooling cycles. The samples were heated from 298.15 K to 493.15 K at 10 K min^−1^ under a nitrogen flow. Subsequently, the samples were cooled back to 298.15 K and kept isothermally for 5 min. *T*_g_ was determined from the second cycle in the TRIOS v5.1.1.5 software, and it was defined as the midpoint of the temperature range where changes to the heat capacity occur.

Thermogravimetric analysis for EHL was performed using a TGA 5500. The EHL sample was placed in a platinum pan and heated under a nitrogen flow (25 ml min^−1^) at 5 K min^−1^ from 303.15 K to 850 K. DSC and TG calibration and procedures are described in our previous work.^22^

### 2.4. Column preparation and IGC measurements

The column preparation and packing method used in this work is described in our research group's previous work.^23^ We prepared a packed stainless-steel column with a length of 1 m and a diameter of 1.59 mm for EHL. The support material (Chromosorb WAW-DMCS 100-120) was coated with 14.49 wt% EHL. The coating was prepared by mixing the dried EHL, Dimethylformamide, and the support material. A BÜCHI Rotavapor R200 was used to prepare the EHL coating material, with rotary evaporation ensuring uniform spreading onto the support surface. The prepared coating material was put in a drying oven for 7 days. Later, the column was filled with the coating material. The Agilent 6890N gas chromatograph equipped with a thermal conductivity detector (TCD) was used for the IGC method to determine the *γ*^∞^ and *V*_g_ for the probe solvents. The equipment description and validation are described in previous work from our research group.^23^ Before any measurements, the column was conditioned at 438.15 K for 8 hours under helium gas flow. In this work, we use 0.2 µL injections with 1 : 1 and 15 : 1 split ratios, where the injection volumes were small enough to reach the infinite-dilution region. Operating conditions were established from preliminary measurements, and the experiments were carried out at three different column temperatures of 408.15, 423.15, and 438.15 K. The injection and detector were set at 523.15 K. Each probe solvent was injected three times, and the average retention time was used to calculate the *γ*^∞^ and *V*_g_. The stability of measurements for *γ*^∞^ and the *V*_g_ from the carrier gas flow rate was checked for four probe solvents: nonane, decane, methanol, and dimethyl sulfoxide. The measurement flow rates were between 5.5 and 10.3 mL min^−1^ at a column temperature of 438.15 K.

### 2.5. Gravimetric solubility of EHL in organic solvents

Gravimetric solubility experiments were conducted in isothermal glass-cell apparatuses at 298.15 K for 22 solvents. The equipment description and validation are described in previous work from our research group.^22^ EHL was mixed with each solvent in sealed glass-cell apparatuses, and the solution was magnetically stirred for 4 hours and allowed to settle for 24 hours. Equilibrium was confirmed when no significant change in EHL concentration was observed beyond these durations. The initial concentration of EHL in the solvent was 20 g L^−1^, as reported by Dastpak *et al.*^24^ After reaching equilibrium, samples were extracted with syringes coupled to a 0.45 µm-pore-size polypropylene filter. The filtered samples were stored in a drying oven at 333.15 K for 7 days, and the sample masses were monitored periodically until constant mass was achieved. The % of initially added EHL dissolved in the gravimetric solubility experiment was calculated based on eqn (1).1
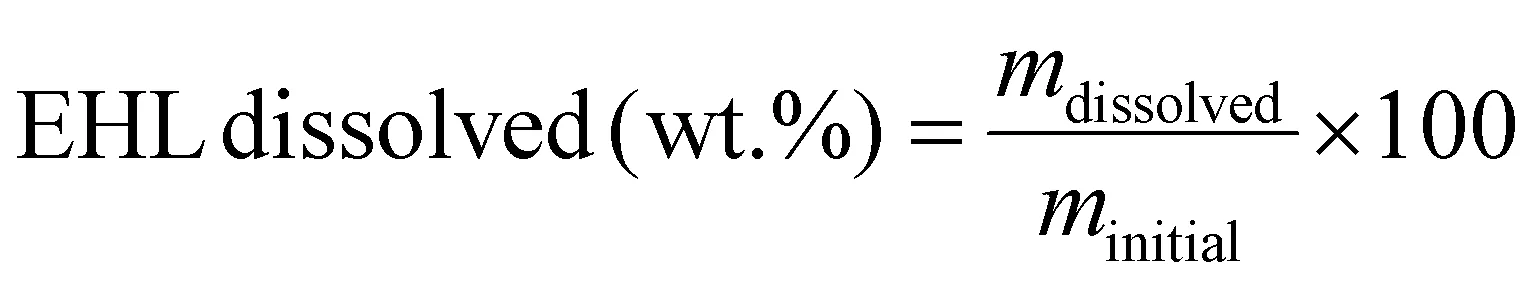
where *m*_initial_ is the initial mass of EHL, *m*_dissolved_ is the mass dissolved, and EHL dissolved (wt%) is the % dissolution of EHL in solvents. Solvents were classified as good (1) or poor (0) using a solubility threshold of 70%, following Novo and Curvelo.^25^ The *R*_0_ of the EHL Hansen sphere was determined as the radius minimizing the number of misclassified solvents relative to the Hansen distance *R*_a_ calculated from the IGC-derived EHL center.

## 3. Theoretical calculations

### 3.1. Determination of infinite dilution activity coefficient and specific retention volume

*γ* ^∞^ and *V*_g_ between the probe solvent and EHL (stationary phase) can be determined using a mathematical formulation developed by Everett^26^ and Cruickshank *et al.*^27^*γ*^∞^ is calculated based on eqn (2).2
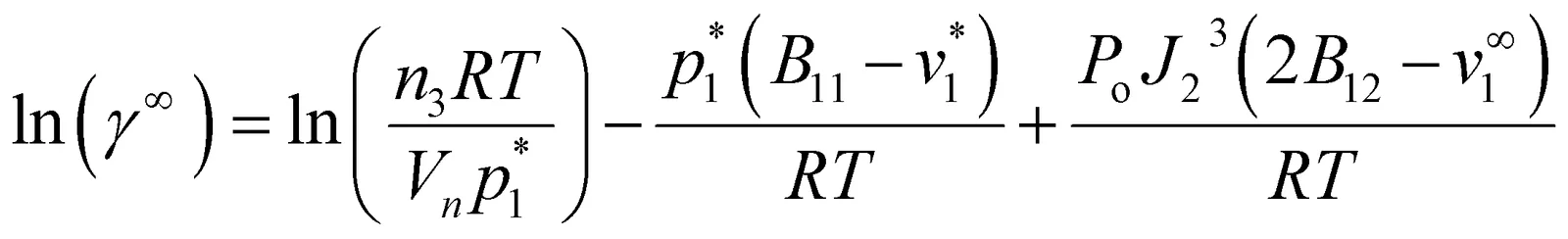
Where *R* = gas constant, *n*_3_ = mole of EHL, *T* = column temperature, *V*_n_, p^*^_1_, *B*_11_, v^*^_1_ is the net retention volume, saturated vapor pressure of the probe solvent, second virial coefficient of the probe solvent, and liquid molar volume of the probe solvent, respectively. *P*_o_ = outlet pressure, *J*_2_^3^ = pressure correction, *B*_12_ = mixed second virial coefficient of the probe solvent and carrier gas, and *v*^∞^_1_ = partial molar volume of solvent probe at infinite dilution in the stationary phase. In this work, *v*^∞^_1_ = v^*^_1_ for probe solvents. The *B*_11_, v^*^_1_, and p^*^_1_ variables were obtained from the DIPPR database^28^ and *B*_12_ is calculated according to literature.^29,30^ The parameters for *B*_11_, v^*^_1_, and p^*^_1_ are provided in the SI, Tables S2, S3, and S4, respectively. Due to a lack of experimental data for dihydrolevoglucosenone and dimethyl isosorbide, the Tsonopoulos correlation was used to estimate *B*_11_ of these probe solvents.^29^ The extended Antoine equation was used to fit the saturated vapor pressure data for dimethyl isosorbide obtained from Durand.^31^ This was due to no vapor pressure data being available in the DIPPR database.

The *J*_2_^3^ is determined as below:3
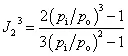
where *p*_i_ is the inlet pressure. The net retention volume of probe solvents (*V*_n_) is calculated as shown in eqn (4).

4 *V*_n_ = (*J*_2_^3^)^−1^*U*_o_(*t*_r_ − *t*_g_)

Where *t*_r_ and *t*_g_ are the retention times of the probe solvent and non-retained gas, respectively. *U*_o_ is the outlet flow, and it is corrected to match the column conditions. The Van't Hoff model is used to fit the *γ*^∞^ experimental data. Eqn (5) represents the fitted parameters for the Van't Hoff model.5
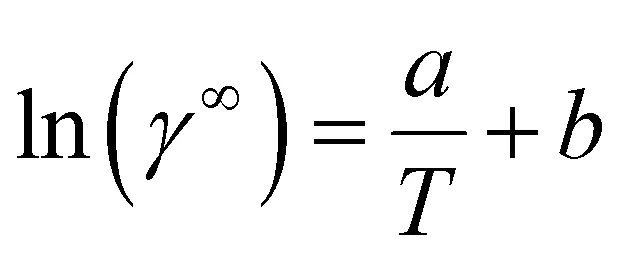
where *a* and *b* are fitted parameters.

The specific retention volume characterizes the elution behavior of the probe solvents in the column; eqn (6) represents the *V*_g_.^32,33^6
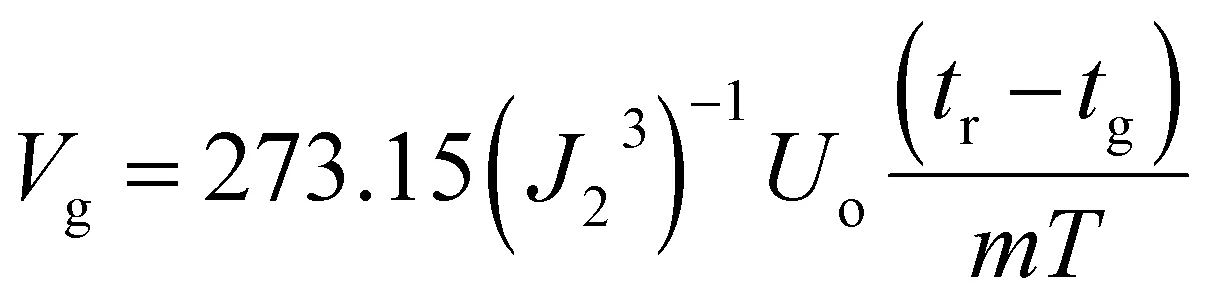
where *m* is the mass of EHL in the column packing.

### 3.2. Flory–Huggins interaction parameter and Hansen solubility parameters

According to the Flory–Huggins theory, the interaction parameter *x*^∞^_13_ for the probe solvent on the solid surface can be calculated as follows:^34^7
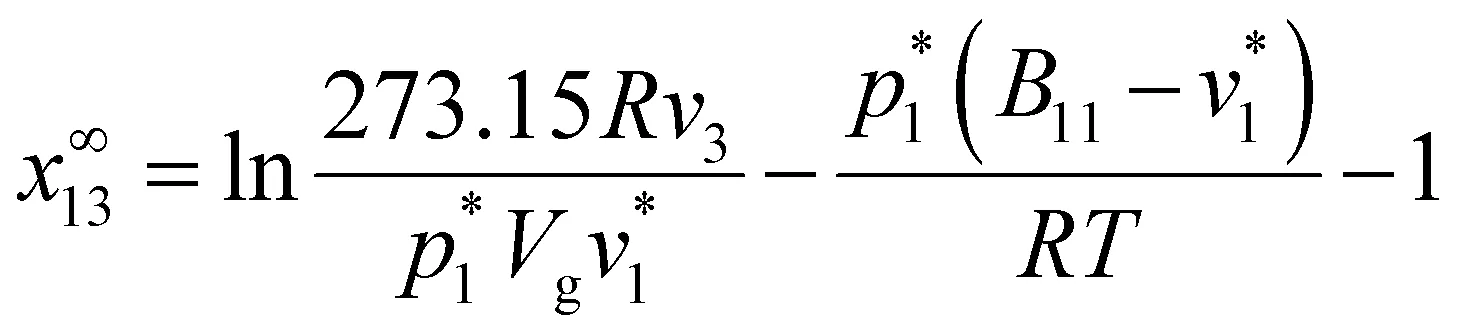
where *v*_3_ is the specific volume of the polymer. Flory–Huggins interaction parameter less than 0.5 was classified as a thermodynamically favorable solvent, whereas higher values indicate limited compatibility of the stationary phase and probe solvent.

The Hildebrand solubility parameter is derived from regular solution theory and does not account for polar or hydrogen-bonding interactions. Hansen addressed this limitation by resolving the total cohesive energy into three-dimensional solubility parameters, which are given in eqn (8).^14^

8 *δ*_t_^2^ = *δ*_D_^2^ + *δ*_P_^2^ + *δ*_H_^2^

where *δ*_t_, *δ*_D_, *δ*_P_, and *δ*_H_ are the total solubility parameter, dispersion solubility parameter, polar solubility parameter, and hydrogen bonding solubility parameter, respectively.

In this work, the experimental column temperatures for IGC are below the glass transition temperature of EHL, so the solubility parameter model developed by DiPaola-Baranyi and Guillet^35^ is therefore not directly applicable to this work, as it is based on the assumption that the probe solvent is absorbed into, and equilibrated with, the bulk of the stationary phase. Instead, the surface adsorption model by Snyder–Karger^36^ was used in this work. Voelkel *et al.*^37^ later developed a method for determining HSP on a solid surface using IGC, based on the Snyder–Karger adsorption model. The energy of adsorption of the probe solvent on the surface of EHL can be calculated based on eqn (9).

9 −Δ*E*^A^ = v^*^_1_(*δ*_D1_*δ*_D2_ + *δ*_P1_*δ*_P2_ + *δ*_H1_*δ*_H2_)

where Δ*E*^A^ is the adsorption energy; the indices 1 and 2 denote the probe solvent and EHL, respectively. The adsorption energy is determined from the temperature dependence of the *V*_g_ using eqn (10).10
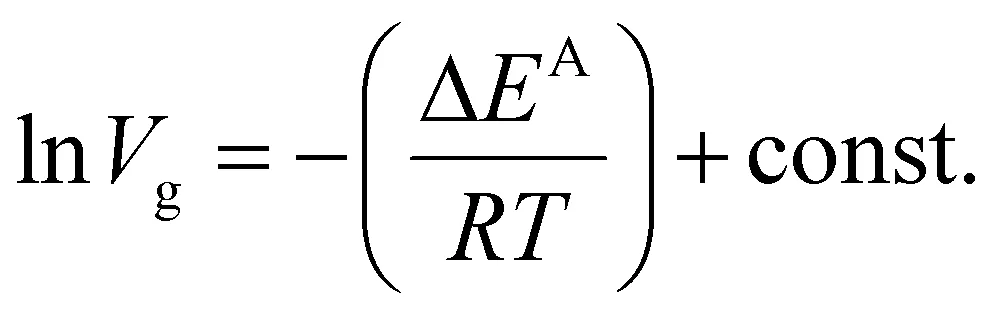


HSPs for EHL were obtained from eqn (9) and (10), following the approach by Voelkel *et al.*^37^ and Ramanaiah *et al.*^38^

Temperature-dependent HSPs for probe solvents were calculated at the column temperature based on the correlation developed by Hansen and Beerbower.^14,39^ The correlations are given in eqn (11)–(13):11
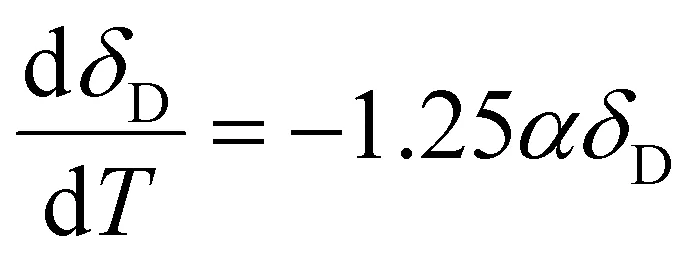
12
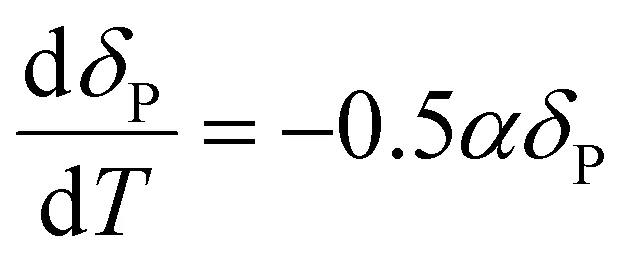
13
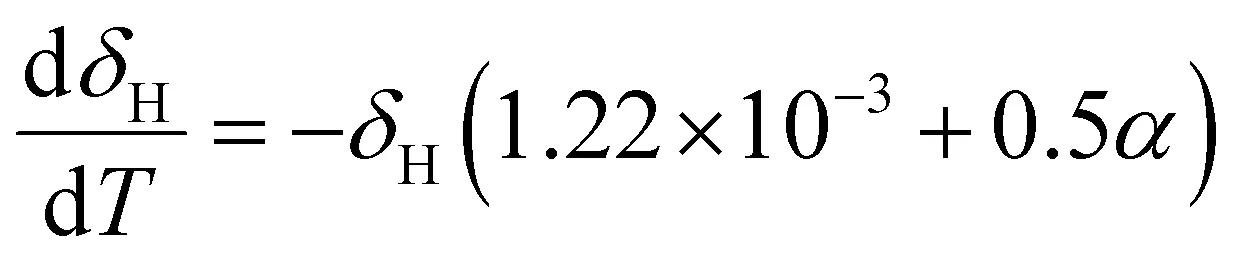
where *α* (K^−1^) is the coefficient of thermal expansion. HSP for probe solvents at column temperatures are given in the SI, Table S6.

### 3.3. EHL Hansen solubility sphere

The relative energy difference was used to determine the miscibility of EHL in solvents.14
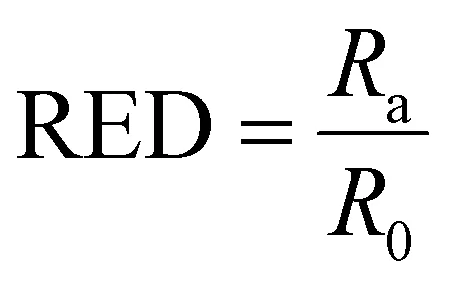

15 *R*_a_^2^ = 4(*δ*_D1_ − *δ*_D2_)^2^ + (*δ*_H1_ − *δ*_H2_)^2^ + (*δ*_P1_ − *δ*_P2_)^2^

where RED is the relative energy difference. Each solvent occupies a fixed point in the three-dimensional space, while the EHL is represented by a sphere of radius *R*_0_. The affinity of a given solvent for the EHL is quantified by the distance between the solvent and the EHL center in Hansen space. A RED value of ≤1.0 indicates a good solvent, while a RED value higher than 1 implies a poor solvent.^14,39^

## 4. Results and discussion

### 4.1. Molecular weight and thermal analysis of EHL

The number average (*M*_n_), weight average (*M*_w_), and PDI of EHL were determined *via* GPC, following the procedure from Dastpak *et al.*^24^ The EHL chromatogram is shown in the SI, Fig. S1. The *M*_w_ and *M*_n_, and PDI of EHL were 6245.00 g mol^−1^, 885.70 g mol^−1^, and 7.05, respectively. A high polydispersity index indicates a broad molecular weight distribution of EHL. The molecular weight of EHL in this work is used to determine *γ*^∞^ in eqn (2).

The thermograms of DSC and TG for EHL are reported in the SI, Fig. S2. DSC thermogram showed a single glass transition region with an inflection point at 450 K. This value is within the range of reported *T*_g_ for lignin.^40,41^ Zhou *et al.*^41^ reported *T*_g_ of 462.5 K for corn stover EHL. Different *T*_g_ of EHL are due to molecular weight distribution, different types of functional groups, extraction processes, and impurities in the EHL sample.^40,42^ No melting endotherm was observed, and the DSC thermogram showed no secondary events or shape distortion typically associated with thermal decomposition, confirming that the EHL sample was amorphous.

The TG thermogram shows an initial mass loss of 6–7% between 305 and 420 K, corresponding to water content in EHL. The % mass between 420 and 470 K remains substantially the same. However, above 500 K, the EHL sample undergoes continuous mass loss, indicating thermal decomposition. The EHL IGC experiments were conducted below the *T*_g_ where the probe solvent adsorbs at the surface of EHL, and the solubility parameters reported in Section 4.3 are attributed to adsorption on the solid surface of the EHL. In the case of dimethyl sulfoxide, dimethylformamide, and *γ*-valerolactone, the IGC experiments were also performed above *T*_g_ in the bulk absorption region. The results are reported in Section 4.2.

### 4.2. Infinite dilution activity coefficient and specific retention volume

Experimental *γ*^∞^ and *V*_g_ for 31 probe solvents in EHL at 408.15, 423.15, and 438.15 K are summarized in Table 1. For nearly all probe solvents, the *γ*^∞^ and *V*_g_ decrease with an increase in temperature consistent with the temperature dependence of probe solvent reported in the previous IGC study for EHL.^43^ This trend is generally associated with the exothermic nature of probe solvent sorption.^44,45^ Lower *γ*^∞^ denote thermodynamically favorable affinity between the solvent probe and EHL. Dimethyl sulfoxide has the lowest *γ*^∞^ values (0.11–0.07) across the experimental temperature range, followed by dimethylformamide. In contrast, the long-chain aliphatic hydrocarbons displayed the highest *γ*^∞^ values due to poor affinity of the probe solvent to the EHL structure. The *γ*^∞^ trends are in line with Ni *et al.*^10^ results for alkali lignin and Zhao *et al.*^43^ results for EHL, where hydrocarbons like *n*-hexane have poor affinity compared to polar aprotic solvents like acetone. Higher *γ*^∞^ for non-polar probe solvents and lower *γ*^∞^ for strong aprotic probe solvents in EHL is due to the abundance of hydroxyl, methoxyl, and carbonyl groups that favor interactions with polar, hydrogen-bond-accepting solvent probes over non-polar aliphatic hydrocarbons.^46,47^

**Table 1:** Average experimental *γ*^∞^ and *V*_g_ for 31 probe solvents in EHL^a^

Temperature	408.15 K		423.15 K		438.15 K	
Probe solvent	*γ* ^∞^	*V*_g_ (cm^3^ g^−1^)	*γ* ^∞^	*V*_g_ (cm^3^ g^−1^)	*γ* ^∞^	*V*_g_ (cm^3^ g^−1^)
Nonane	50.00	0.51	48.86	0.35	44.13	0.27
Decane	52.25	0.95	51.44	0.62	48.63	0.44
Undecane	56.80	1.68	56.15	1.04	54.09	0.69
Dodecane	59.87	3.05	59.81	1.79	58.87	1.12
Hexadecane	74.04	30.27	73.44	14.96	70.20	8.32
Methanol	0.43	4.97	0.38	3.58	0.37	2.26
Ethanol	0.73	3.64	0.64	2.87	0.64	2.08
1-Propanol	2.46	2.06	1.71	2.01	1.29	1.87
1-Pentanol	11.25	1.59	7.19	1.57	4.75	1.57
*o*-Xylene	19.56	1.08	18.54	0.77	14.05	0.71
*p*-Xylene	18.59	0.97	16.25	0.76	11.57	0.75
Dimethylformamide	0.19	138.66	0.19	92.24	0.18	67.51
*γ*-Valerolactone	2.67	47.94	1.64	47.74	1.06	46.98
Benzene	4.96	0.83	4.74	0.65	4.58	0.51
d-Limonene	30.99	1.65	30.98	1.08	29.50	0.77
α-Pinene	35.25	0.81	34.73	0.56	32.72	0.42
Benzonitrile	3.27	24.62	2.02	24.87	1.33	24.50
Toluene	13.09	0.67	9.44	0.67	7.04	0.66
Tetrahydrofuran	2.01	1.43	1.97	1.10	1.94	0.87
Acetone	1.38	1.63	1.06	1.64	0.89	1.51
Methyl acetate	2.14	1.05	1.63	1.05	1.31	1.04
Ethyl acetate	2.75	1.33	2.74	0.99	2.68	0.78
Propyl acetate	6.83	1.00	6.80	0.72	6.57	0.55
Butyl acetate	18.95	0.69	14.94	0.60	10.54	0.60
Cyclohexane	23.72	0.18	19.35	0.17	17.24	0.15
Cyclohexene	27.69	0.17	21.02	0.16	18.80	0.14
Dimethyl sulfoxide	0.11	1.3 × 10^3^	0.09	577.32	0.07	304.14
Ethylbenzene	19.70	0.86	16.46	0.71	12.10	0.69
Dimethyl carbonate	1.81	2.75	1.75	2.06	1.38	1.94
Dimethyl isosorbide	20.81	19.92	14.04	15.95	8.33	15.33
Dihydrolevoglucosenone	14.07	17.81	8.40	17.27	5.01	17.24

a *U*_rel_(*γ*^∞^) = 10% with 95% coverage factor *K* = 2.

Fig. 1 represents the experimental *γ*^∞^ data for EHL in (a) aliphatic hydrocarbons, (b) polar aprotic solvents, (c) alcohols, and (d) bio-based solvents. The Van't Hoff model was used to fit the *γ*^∞^ experimental data. The correlations for the model are provided in the SI, Table S5. A systematic trend in *γ*^∞^ can be seen in Fig. 1 where an increase in carbon number for alkanes, acetates, and alcohols leads to poor affinity of probe solvents with EHL. A similar trend for alkanes and alcohols based on carbon number can be seen in Mustafa.^44^ Since the column temperature is below the *T*_g_, the stability of measurements for *γ*^∞^ as a function of flow rate for probe solvents was determined at 438.15 K. Fig. S3(a) and (b) of the SI show that the *γ*^∞^ for nonane, decane, methanol, and dimethyl sulfoxide are within 5% across flow rates of 5.5–10.3 mL min^−1^, with no significant dependence on probe solvent. The *γ*^∞^ values derived in this work are below the glass transition temperature and therefore reported as apparent quantities, reflecting probe–surface rather than probe–bulk interactions.

**Fig. 1:**
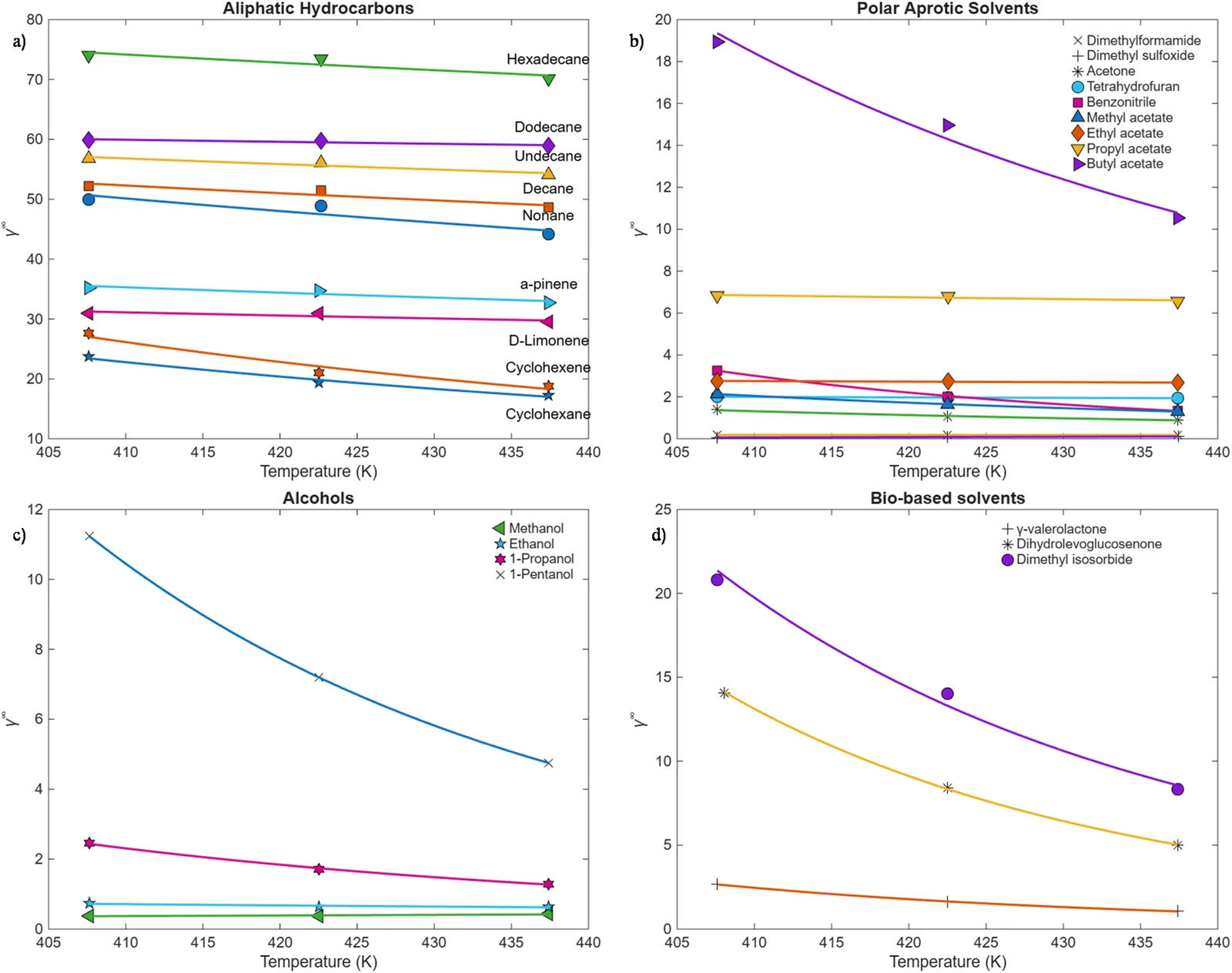
Experimental *γ*^∞^ data for EHL with (a) aliphatic hydrocarbons, (b) polar aprotic solvents, (c) alcohols, and (d) bio-based solvents. Lines = Van't Hoff model.

In the case of *V*_g_, the same trend can be seen, where for dimethyl sulfoxide the *V*_g_ is more than four orders of magnitude higher than *V*_g_ for cyclohexene. Higher *V*_g_ for polar aprotic solvents such as dimethyl sulfoxide and dimethylformamide is due to strong dipole–dipole interactions and strong hydrogen-bond-acceptor capabilities.^22^ This leads to a large disparity in retention times between the hydrogen-bond-accepting and dipolar solvents and non-polar aliphatic and alicyclic hydrocarbons. Dimethyl sulfoxide, dimethylformamide, dihydrolevoglucosenone, dimethyl isosorbide, *γ*-valerolactone, and benzonitrile exhibited the highest retention, whereas cyclohexane, cyclohexene, and the aromatic hydrocarbons were retained the least. For alkanes and acetates, the value of *V*_g_ increased with increasing molecular weight (Table 1), indicating that the molecular weight of probe solvents can influence the interaction with lignin.^43^ The *V*_g_ obtained for EHL in this work were compared with those reported by Zhao *et al.*^43^ at 423.15 K in the case of cyclohexane, benzene, methanol, ethanol, 1-propanol, acetone, and tetrahydrofuran. The comparison between the *V*_g_ are provided in Fig. S4 of the SI. Overall, the *V*_g_ values showed good agreement with Zhao *et al.*,^43^ although some differences were observed depending on the solvent. These differences may be attributed to variations in lignin characteristics, experimental conditions, and interactions between EHL and the respective probe solvents.

ln *V*_g_ was plotted against 1000/*T* for 15 selected probe solvents in Fig. 2. The retention diagrams were linear for the probe solvents, indicating no phase change in the EHL over the temperature range of 408.15–438.15 K. The retention diagrams for the probe solvents were attributed to adsorption on the solid surface of the EHL, since the experimental temperatures were below the glass-transition range. For dimethyl sulfoxide, *γ*-valerolactone, and dimethylformamide, the retention diagram was extended to 473.15 K. This is due to the relatively high *V*_g_ of these probe solvents and their stronger retention allowed more reliable comparison of *V*_g_ and *γ*^∞^ at higher column temperatures of 463.15–473.15 K. Table 2 presents measured *γ*^∞^ and *V*_g_ in the bulk-absorption regions, where the reported *γ*^∞^ are intrinsic quantities rather than apparent quantities. Both *γ*^∞^ and *V*_g_ decrease with an increase in temperature. Fig. 3 shows the probe solvent profile for the retention diagram as it passes through the EHL's glass transition region. The resulting retention diagram exhibits the characteristic Z-shaped profile associated with the glass transition of a polymeric stationary phase.^44,48^ Three regions were distinguished: surface adsorption, glass transition region (determined *via* DSC and IGC), and bulk absorption. Below 440 K, ln *V*_g_ decreases linearly with 1/T, which corresponds to surface adsorption, while the linear trend at the high-temperature part of the Z-shaped retention diagram corresponds to bulk absorption in the stationary phase.^48^

**Fig. 2:**
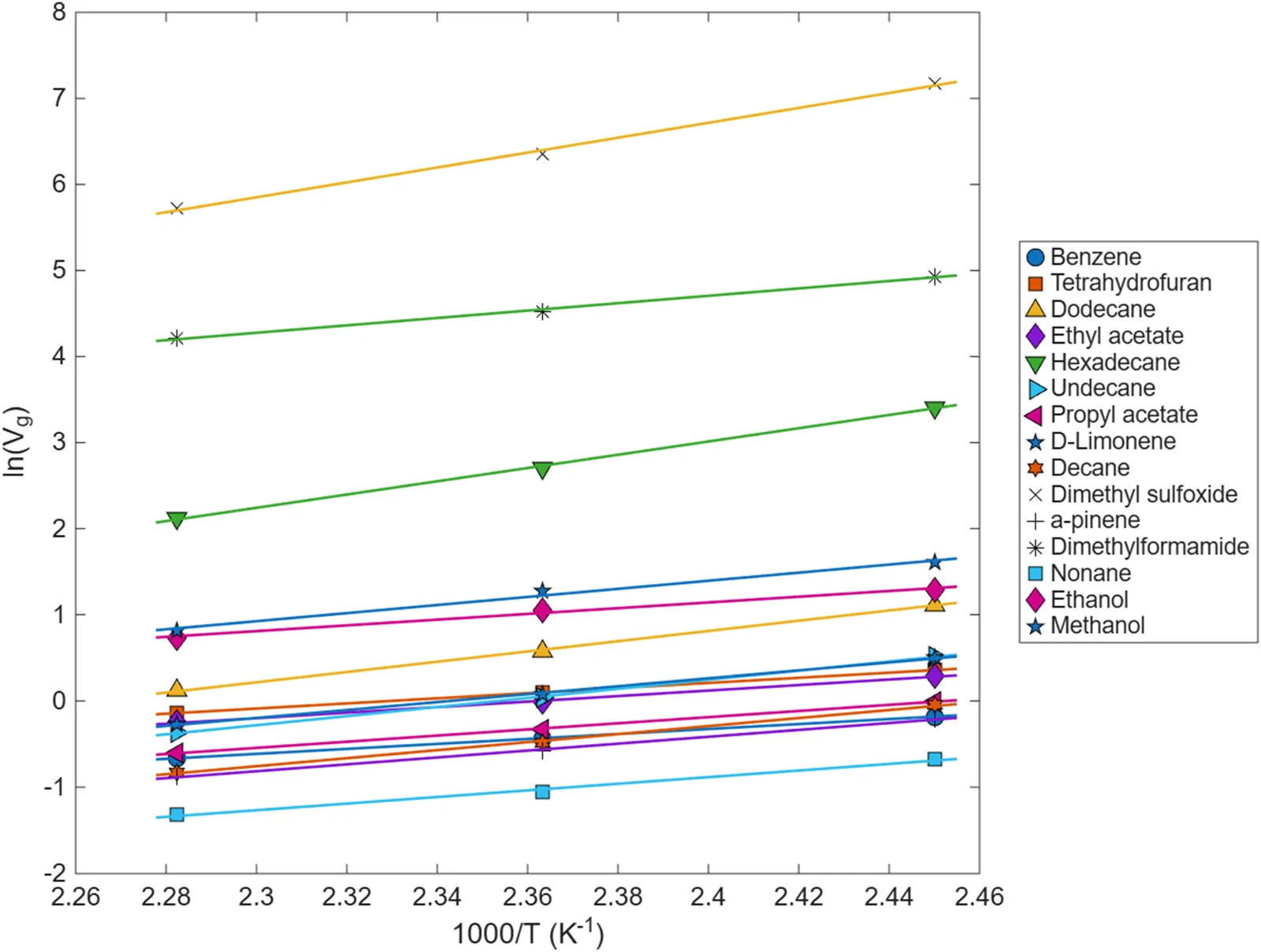
Retention diagram of 15 probe solvents over the temperature range of 408.15–438.15 K for EHL.

**Table 2:** Average experimental *γ*^∞^ and *V*_g_ for dimethylformamide, *γ*-valerolactone, and dimethyl sulfoxide in the bulk absorption region^a^

Temperature	463.15 K		468.15 K		473.15 K	
Probe solvent	*γ* ^∞^	*V*_g_ (cm^3^ g^−1^)	*γ* ^∞^	*V*_g_ (cm^3^ g^−1^)	*γ* ^∞^	*V*_g_ (cm^3^ g^−1^)
Dimethylformamide	0.17	20.47	0.17	18.07	0.16	14.99
*γ*-Valerolactone	1.00	25.15	0.99	22.52	0.99	19.71
Dimethyl sulfoxide	0.06	106.80	0.06	89.35	0.05	72.03

a *U*_rel_(*γ*^∞^) = 10% with 95% coverage factor *K* = 2.

**Fig. 3:**
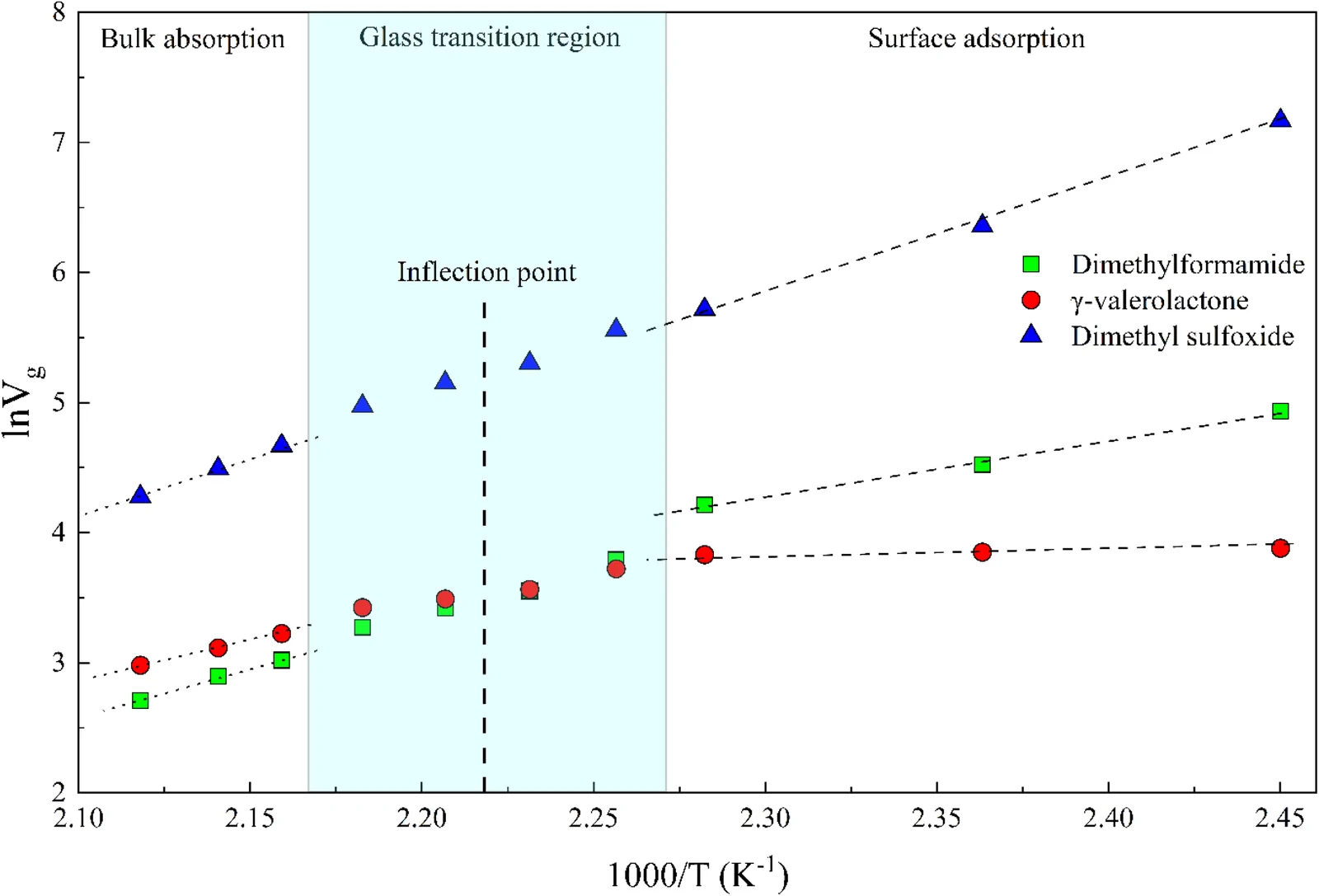
Change in logarithmic specific retention volume over the temperature range of 408.15–473.15 K for EHL.

### 4.3. Flory–Huggins interaction parameter and HSPs of EHL

Flory–Huggins interaction parameters for probe solvents are provided in SI Table S7. *x*^∞^_13_ lower than 0.5 are quantified as good solvents, whereas values between 0.5 and 5 are quantified as poor solvents.^43^*x*^∞^_13_ values range from −0.82 for dimethyl sulfoxide to 4.56 for nonane. *x*^∞^_13_ decrease with increasing temperature and are reported as apparent quantities to compare the affinity of probe solvents in EHL. Dimethyl sulfoxide and dimethylformamide are the only probes with negative *x*^∞^_13_ due to high dissolution and their capacity to accept hydrogen bonds from EHL hydroxyl groups. Aliphatic hydrocarbons have values greater than 4 because of their poor affinity for EHL.

The Hildebrand and Hansen solubility parameters were determined at different temperatures by eqn (8–10). The Snyder–Karger adsorption model was used to calculate the HSPs based on the energy of adsorption of the probe solvent on the EHL surface.^37^ Table S8 of the SI contains the energy of adsorption of probe solvents on the surface of EHL. All values are negative, confirming that adsorption is exothermic, with dimethyl sulfoxide having the lowest value of −72.09 kJ mol^−1^. For aliphatic hydrocarbons, the adsorption energy decreases with increasing carbon number, from −31.98 to −64.26 kJ mol^−1^. This is due to dispersion interactions between the EHL surface and the probe solvent.^49^ HSPs of EHL were obtained from regression of eqn (9), where the HSPs for probe solvents at different temperatures were determined by eqn (11)–(13). The HSP values for probe solvents at different temperatures are given in Table S6 of the SI. Hildebrand solubility parameters for EHL were calculated based on eqn (8). As shown in Table 3, the increase in temperature from 408.15 to 438.15 K shows a decrease in Hildebrand and Hansen solubility parameters. The Hildebrand solubility parameter of EHL varies within the range of 20.71–21.48 (MPa^1/2^). To determine the EHL at 298.15 K, the method described by Zhao *et al.*^43^ was used to extrapolate HSP from the relationship curve provided in the SI, Fig. S5.

**Table 3:** Hildebrand and Hansen solubility parameters of EHL at varied temperatures

*T* (K)	*δ*_D_ (MPa^1/2^)	*δ*_P_ (MPa^1/2^)	*δ*_H_ (MPa^1/2^)	*δ*_t_ (MPa^1/2^)
408.15	18.11	8.82	7.44	21.48
423.15	17.97	8.40	7.36	21.15
438.15	17.83	7.69	7.21	20.71
298.15^a^	19.15	13.01	8.30	24.58

a Extrapolation using the method of Zhao *et al.*^43^

EHL HSPs in this work at 298.15 K were compared with HSPs for other types of lignin in Table 4. The HSPs values in this work specifically correspond to EHL obtained from corncob conversion to ethanol. According to Table 4, the HSPs of lignin vary considerably depending on the lignin type. The differences in values are due to variations in chemical structure, molecular weight, degree of condensation, biomass feedstock, and treatment processes.^50^ In terms of chemical structure, different groups such as phenolic, aliphatic hydroxyl, methoxy, and carbonyl groups alter dispersion, polarity, and especially the hydrogen-bonding solubility parameter.^7,51^ In the case of the same type of lignin source, different treatment processes can cause ether bond cleavage, condensation, and oxidation reactions that modify lignin functional group distribution and consequently their HSP values.^25,52^

**Table 4:** Solubility parameters of different types of lignin at 298.15 K

Lignin type	*δ*_D_ (MPa^1/2^)	*δ*_P_ (MPa^1/2^)	*δ*_H_ (MPa^1/2^)	*δ*_t_ (MPa^1/2^)	Ref.
EHL	19.15	13.01	8.30	24.58	This work
EHL	—	—	—	23.81	43
Milled wood lignin	21.90	14.10	16.90	31.05	53
Alkali lignin	17.21	12.35	12.33	24.50	10
Organosolv lignin	21.42	8.57	21.80	31.70	25
Sugar cane bagasse lignin	10.50	4.17	10.70	15.50	51
Hardwood kraft lignin	16.70	13.50	11.30	24.30	54

### 4.4. EHL gravimetric solubility experiments and determination of *R*_0_

The EHL gravimetric solubility results for 22 pure solvents were measured at 298.15 K. Fig. 4 represents the EHL dissolved (wt%) and ln *γ*^∞^ results of EHL in 22 pure solvents. Each gravimetric solubility experiment was performed twice, and the mean value was reported. Fig. 4 represents a qualitative comparison between the experimental EHL dissolved (wt%) and the ln *γ*^∞^ for the respective solvent, where the ln *γ*^∞^ increases with decreasing EHL dissolved (wt%). SI Table S9 contains gravimetric solubility results, whereas the visual representation of dissolved EHL in solvents is given in Fig. S6 of the SI. The samples were collected using a syringe coupled to a 0.45 µm pore-size polypropylene filter, and the darker coloration in Fig. S6 qualitatively corresponds to a higher dissolved lignin fraction. In Fig. 4, the highest EHL dissolved (wt%) was observed for dipolar aprotic solvents such as dimethyl sulfoxide and dimethylformamide, due to their strong hydrogen-bond acceptor capacity, lack of hydrogen-bond donor capacity, and high polarity. *γ*-Valerolactone, acetone, and tetrahydrofuran likewise dissolved more than 70% of the EHL, whereas non-polar aliphatic and aromatic solvents have the lowest EHL dissolved (wt%).^55–57^

**Fig. 4:**
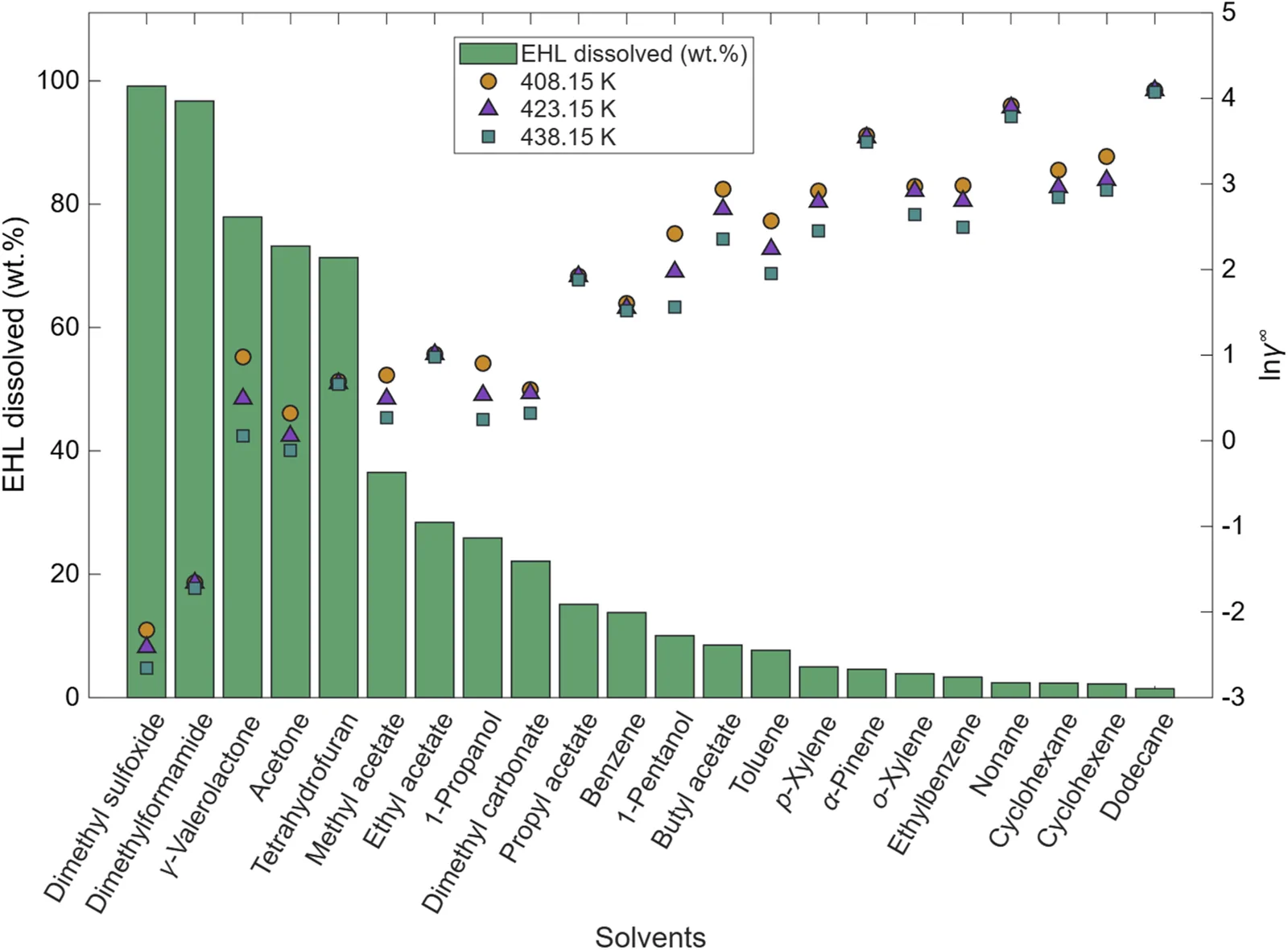
Gravimetric solubility and ln *γ*^∞^ results of EHL in 22 pure solvents. The gravimetric solubility experiments were conducted at 298.15 K.

Based on the gravimetric solubility results, Novo and Curvelo^25^ procedure for optimizing *R*_0_ was followed. In this approach, it is assumed that the relative energy difference should provide a linear relationship with the lignin solubility. In other words, higher values of RED for a solvent/lignin system should provide lower solubility results.^25^ According to Fig. 5, the best five pure solvents (solubility thresholds higher than 70%) presented RED values lower than 1. The RED values for the remaining 17 solvents are well dispersed across the entire RED range. However, the *R*^2^ value is 0.87, which is moderate for good linear regression. Solubility thresholds of 60% and 80% were also evaluated, but neither improved the linear regression; therefore, the 70% threshold was the best fit. Table S10 of the SI contains fitted *R*_0_ for 60–80% solubility thresholds. *R*_0_ was obtained from the best correlation between RED and EHL dissolved (wt%), and it was found to be 8.98. This value is closer to the alkali lignin value of 8.10 determined by Ni *et al.*^10^ However, the *R*_0_ in this work is lower than the values reported for milled wood lignin (13.7) and sugarcane bagasse organosolv lignin (13.56).^25,51^ This can be due to different types of lignin and the solubility criterion adopted, which differ between the literature sources.

**Fig. 5:**
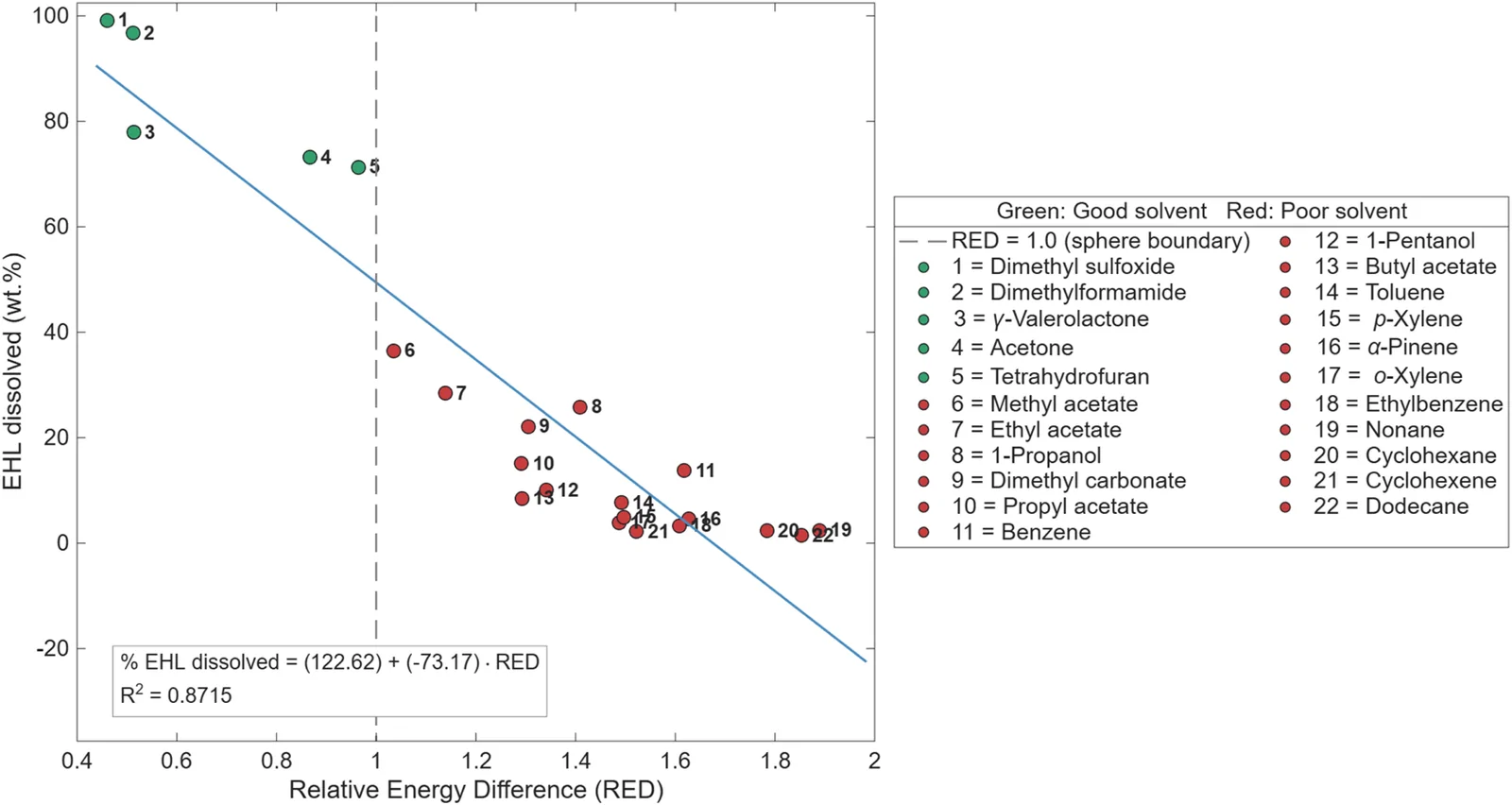
Relative energy difference (RED) for lignin solubility sphere *versus* EHL dissolved (wt%) from solubility measurements at 298.15 K.

### 4.5. Hansen solubility sphere and solvent classification

The Hansen solubility parameters of EHL determined in this work (*δ*_D_ = 19.15, *δ*_P_ = 13.01, and *δ*_H_ = 8.30 MPa^0.5^ at 298.15 K), together with the interaction radius *R*_0_ = 8.98 MPa^0.5^ obtained from the gravimetric solubility measurements (Section 4.4), define the EHL Hansen solubility sphere shown in Fig. 6. Fig. 6(a) represents the three-dimensional Hansen solubility sphere (*δ*_D_*δ*_P_*δ*_H_), and Fig. 6(b–d) represents the two-dimensional projections onto the *δ*_D_–*δ*_P_, *δ*_D_–*δ*_H_, and *δ*_P_–*δ*_H_ planes, respectively. Each solvent is positioned according to its own Hansen coordinates and classified by its RED value. Table S11 of the SI contains RED values and solvent classification for the 31 solvents. Solvents with RED < 1 are classified as good solvents, and those with RED ≥ 1 as poor solvents. According to Fig. 6(a), solvent numbers 1–8 are within the Hansen solubility sphere. Strong polar aprotic solvents like dimethyl sulfoxide and dimethylformamide cluster near the EHL sphere center, which is consistent with high *V*_g_ and low *γ*^∞^ observed for these solvents in the IGC measurements (Section 4.2). On the other hand, non-polar aliphatic and alicyclic hydrocarbons like nonane and cyclohexane are outside the Hansen solubility sphere due to large RED values and poor affinity of these solvents in EHL (coordinate position outside the sphere). The two-dimensional projections, Fig. 6(b–d), further illustrate the solvent distribution around the EHL sphere. In Fig. 6(b and c), the non-polar aliphatic and aromatic hydrocarbons (solvents 21–31) fall into the low-polarity and non-hydrogen-bonding region in contrast to polar aprotic solvents. Furthermore, Fig. 6(d) shows a clear separation between good and poor solvents, indicating that the polar and hydrogen-bonding interactions are dominant factors for solvent classification in EHL. This is consistent with the literature, where Schuerch^58^ and Ni *et al.*^10^ noted that lignin dissolution improves with increasing hydrogen-bonding capacity of the solvent.

**Fig. 6:**
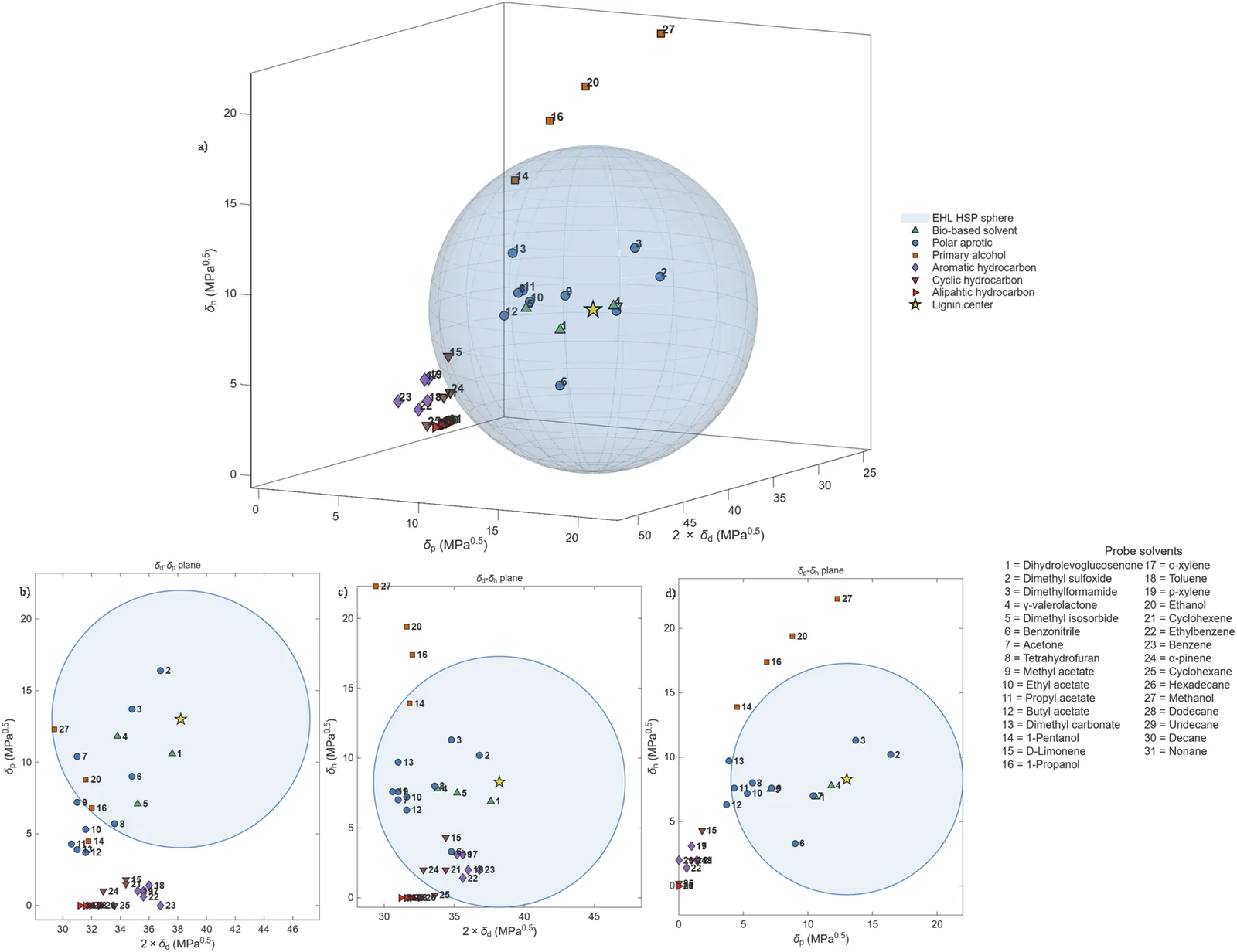
Hansen solubility sphere of enzymatic hydrolysis lignin (EHL) at 298.15 K. (a) Three-dimensional representation in Hansen space (*δ*_D_*δ*_P_*δ*_H_), and (b–d) two-dimensional projections onto the *δ*_D_–*δ*_P_, *δ*_D_–*δ*_H_, and *δ*_P_–*δ*_H_ planes, respectively.

## 5. Conclusions

In this study, *V*_g_ and Hansen solubility parameter theory were combined to calculate the surface properties of EHL *via* the Snyder–Karger adsorption model. The *γ*^∞^ and HSPs of EHL were determined by IGC across 31 probe solvents over 408.15–438.15 K, and the interaction radius was fitted using the gravimetric solubility experiments. The HSPs of EHL at 298.15 K were *δ*_D_ = 19.15, *δ*_P_ = 13.01, *δ*_H_ = 8.30, *δ*_t_ = 24.58 MPa^0.5^, together with the interaction radius *R*_0_ = 8.98 MPa^0.5^. The *γ*^∞^ and *V*_g_ indicate that the EHL-probe solvent affinities are strongly influenced by polar and hydrogen-bonding interactions. Polar aprotic probe solvents, such as dimethyl sulfoxide and dimethylformamide, exhibited thermodynamically favorable affinity between the solvent probe and the EHL. In contrast, long-chain aliphatic hydrocarbons displayed the highest *γ*^∞^ values due to poor affinity of the probe solvent to the EHL structure. In the case of dimethyl sulfoxide, dimethylformamide, and *γ*-valerolactone, the IGC experiments were also performed above *T*_g_ in the bulk absorption region. Furthermore, the RED solvent classification agreed with the gravimetric results for 22 solvents examined. Among the bio-based solvents evaluated, dihydrolevoglucosenone, *γ*-valerolactone, and dimethyl isosorbide all fell within the EHL sphere, with *γ*-valerolactone exhibiting the lowest *γ*^∞^ across the bio-based solvents investigated. These results suggest that these solvents are promising bio-based candidates for further evaluation in lignocellulosic biorefineries. Overall, this study provides a quantitative framework for the selection and screening of sustainable solvents for EHL processing and supporting the development of more efficient and environmentally friendly lignin valorization pathways.

## Author contributions

M.O.: writing – review and editing, writing – original draft, visualization, validation, methodology, investigation, formal analysis, conceptualization. J-P.L.: writing – review and editing, visualization, methodology, investigation. R.L.: investigation. A.A.W.: writing – review and editing, investigation. E.A.: writing – review and editing. P.U–K.: writing – review and editing, supervision, resources, methodology, investigation, funding acquisition, conceptualization. V.A.: writing – review and editing, supervision, resources, project administration, methodology, funding acquisition, conceptualization.

## Conflicts of interest

The authors declare that they have no competing interests.

## Supplementary Material

RA-016-D6RA09398A-s001

## Data Availability

All data supporting the findings of this study are provided in the article and the supplementary information (SI). Supplementary information: Probe solvent specifications; coefficients for molar volume, second virial coefficient, and vapor pressure; EHL characterization data; fitted parameters for the Van't Hoff model; Hansen solubility parameters of the probe solvents; Flory–Huggins interaction parameters; energies of adsorption and gravimetric solubility (wt%) of EHL in the solvents. See DOI: https://doi.org/10.1039/d6ra09398a.
