# Supplementary material for: Solubility parameters of enzymatic hydrolysis lignin: a comparative study of inverse gas chromatography and Hansen solubility sphere
Source: RSC Adv. 2026 Oct 27. Online ahead of print. doi: 10.1039/d6ra09398a (PMC13711123; doi:10.1039/d6ra09398a)
Supplement: Supplementary file 1 [file RA-OLF-D6RA09398A-s001.pdf]

Supplementary Information (SI) for RSC Advances.
This journal is © The Royal Society of Chemistry

Solubility Parameters of Enzymatic Hydrolysis Lignin: A Comparative Study of Inverse Gas Chromatography and Hansen Solubility Sphere

Muhammad Ossama, Juho-Pekka Laakso, Robin Luci, Agung Ari Wibowo, Ehsan Asadi, Petri Uusi-Kyyny, Ville Alopaeus*

Aalto University, School of Chemical Engineering, Department of Chemical and Metallurgical Engineering, Research Group of Chemical Engineering, P.O. Box 16100, FI-

00076 Aalto, Finland

*Corresponding author's Email: Muhammad.ossama@aalto.fi

Table S1: Solvents studied in this study, with CAS numbers, suppliers, and purity

Probe solvent	CAS Number	Supplier	Supplier purity %
Nonane	111-84-2	Sigma-Aldrich	99.0
Decane	124-18-5	Sigma-Aldrich	99.0
Undecane	1120-21-4	Sigma-Aldrich	99.0
Dodecane	112-40-3	Sigma-Aldrich	99.0
Hexadecane	544-76-3	Sigma-Aldrich	99.0
Methanol	67-56-1	Sigma-Aldrich	99.9
Ethanol	64-17-5	Anora Oy	99.5
1-Propanol	71-23-8	Sigma-Aldrich	99.7
1-Pentanol	71-41-0	Sigma-Aldrich	99.0
o-xylene	95-47-6	Sigma-Aldrich	97.0
p-xylene	106-42-3	Sigma-Aldrich	99.0
Dimethylformamide	68-12-2	Sigma-Aldrich	99.8
δ-valerolactone	108-29-2	Sigma-Aldrich	98.0
Benzene	71-43-2	Sigma-Aldrich	99.9
D-limonene	5989-27-5	Sigma-Aldrich	97.0
α-pinene	80-56-8	Sigma-Aldrich	98.0
Benzonitrile	100-47-0	Sigma-Aldrich	99.0
Toluene	108-88-3	Sigma-Aldrich	99.9
Tetrahydrofuran	109-99-9	Sigma-Aldrich	99.9
Acetone	67-64-1	Sigma-Aldrich	99.9
Methyl acetate	79-20-9	Sigma-Aldrich	99.5
Ethyl acetate	141-78-6	Sigma-Aldrich	99.8
Propyl acetate	109-60-4	Sigma-Aldrich	99.5
Butyl acetate	123-86-4	Sigma-Aldrich	99.0
Cyclohexane	110-82-7	Sigma-Aldrich	99.5
Cyclohexene	110-83-8	Sigma-Aldrich	99.0
Dimethyl sulfoxide	67-68-5	Sigma-Aldrich	99.9
Ethylbenzene	100-41-4	Sigma-Aldrich	99.8
Dimethyl carbonate	616-38-6	Sigma-Aldrich	99.0
Dimethyl isosorbide	5306-85-4	Sigma-Aldrich	98.0
Dihydrolevoglucosenone	53716-82-8	Sigma-Aldrich	98.0

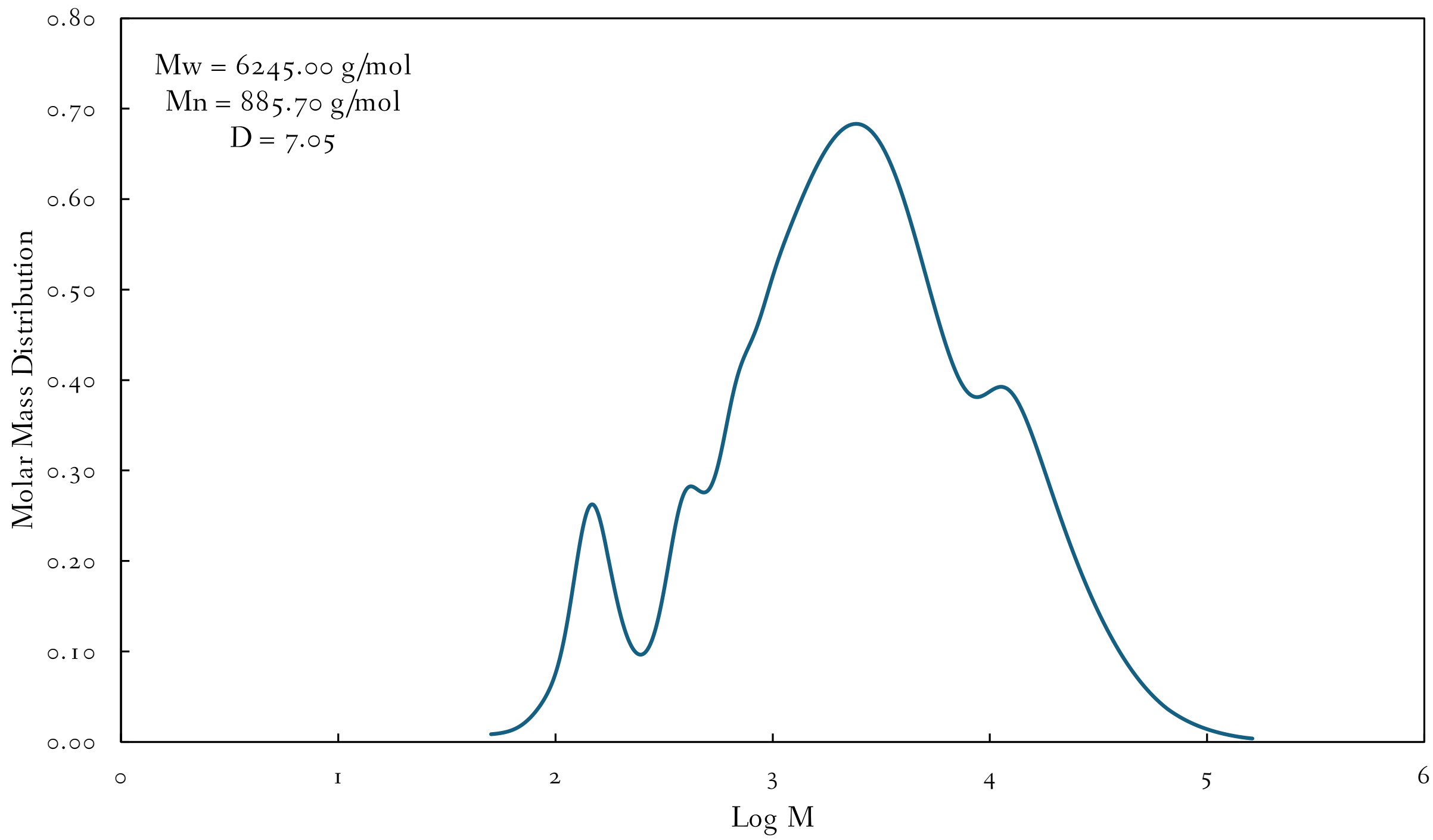

Figure S1: GPC chromatogram of EHL. The calibration was performed using sulfonated polystyrenes

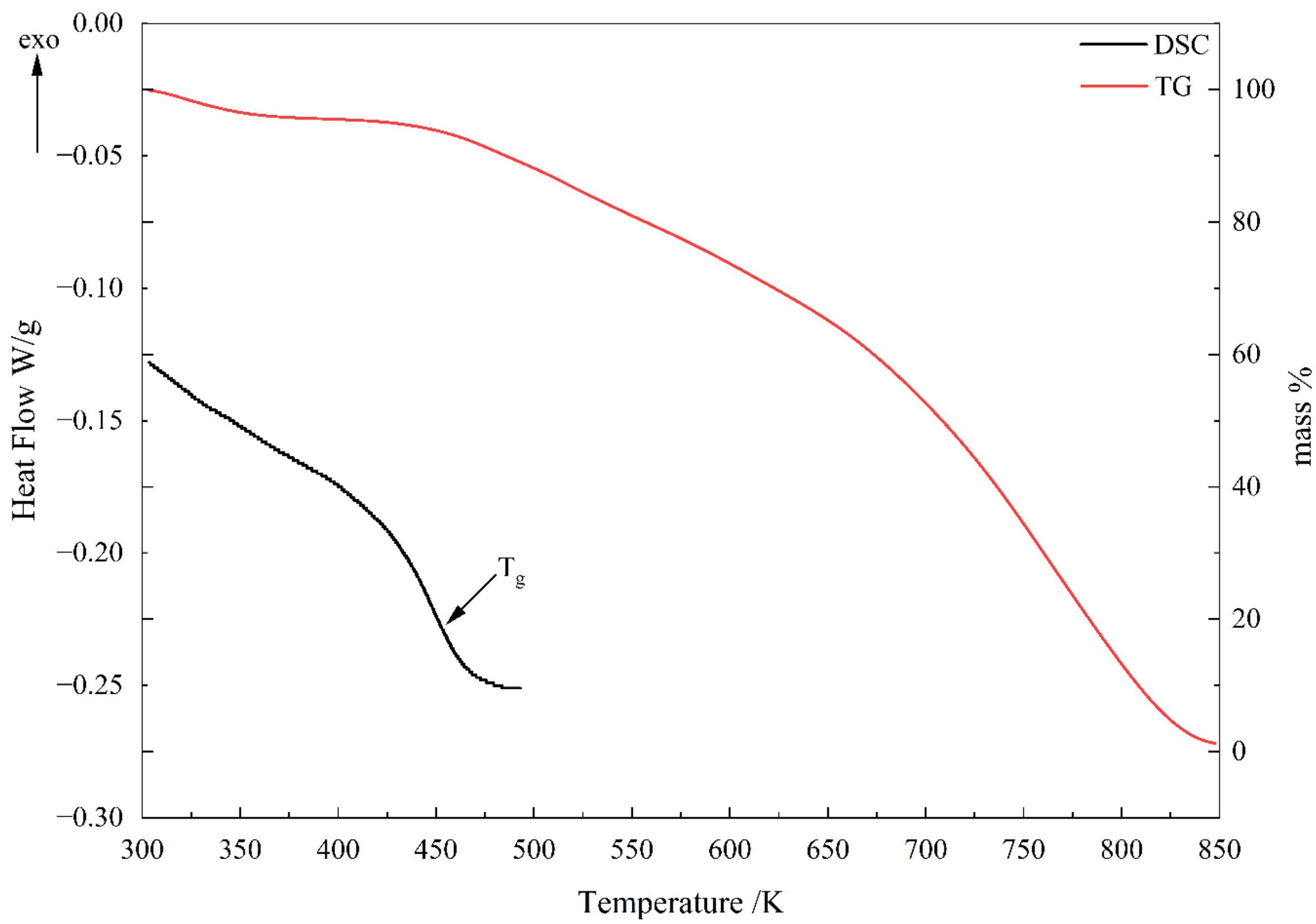

Figure S2: DSC (black line) and TG (red line) of EHL

Table S2: Second virial coefficient for probe solvents

Probe solvent	A	B	C	D	E	Ref
Dimethyl sulfoxide	0.17	-282.99	-60114000	7.55E+20	-3.24E+23	1
Dimethylformamide	0.17	-222.47	-70180000	-8.70E+20	1.53E+23	1
δ-valerolactone	0.21	-299.63	-89927000	-2.93E+20	-2.70E+22	1
Methanol	0.01	-14.40	-20600000	7.60E+19	-4.62E+22	1
Benzonitrile	0.21	-288.80	-105830000	-1.73E+21	3.18E+23	1
Acetone	0.16	-164.67	-20407000	-1.11E+20	1.74E+22	1
Dimethyl carbonate	0.16	-185.32	-28619000	-3.91E+19	1.45E+21	1
Toluene	0.18	-215.67	-43127000	-8.15E+19	1.21E+22	1
o-xylene	0.14	-157.00	-88851000	1.71E+20	-6.70E+22	1
α-pinene	0.33	-465.67	-71740000	-2.27E+19	-3.18E+22	1
Ethyl acetate	0.16	-180.30	-31882000	-4.84E+19	4.11E+21	1
Tetrahydrofuran	0.14	-158.26	-17142000	-6.83E+18	5.67E+20	1
Benzene	0.17	-210.90	-20347000	-3.33E+19	6.70E+21	1
D-limonene	0.33	-424.67	-96460000	-2.31E+20	-1.80E+21	1
Ethylbenzene	0.23	-297.69	-56153000	-3.12E+19	-1.58E+22	1
Butyl acetate	0.23	-269.30	-64410000	-2.34E+20	3.13E+22	1
Ethanol	0.04	-55.70	-12900000	-6.49E+19	-2.58E+22	1
1-Propanol	0.15	-92.47	-32200000	-3.06E+20	2.71E+21	1
Nonane	0.39	-468.60	-66800000	-7.89E+20	1.82E+23	1
Decane	0.48	-588.50	-76060000	-1.61E+21	3.99E+23	1
Undecane	0.58	-727.30	-82940000	-2.96E+21	7.75E+23	1
Dodecane	0.88	-1091.00	-50300000	-5.49E+21	1.50E+24	1
Hexadecane	0.75	-809.99	-445060000	-2.47E+21	1.29E+23	1
1-Pentanol	0.26	-292.21	-27910000	2.71E+20	-1.75E+23	1
p-xylene	0.14	-164.33	-86583000	1.82E+20	-6.76E+22	1
Methyl acetate	0.13	-154.94	-25057000	-1.80E+00	-4.79E+20	1
Propyl acetate	0.19	-211.80	-46040000	-8.00E+19	6.88E+21	1
Cyclohexane	0.18	-187.80	-27480000	-5.48E+19	1.04E+00	1
Cyclohexene	0.18	-222.46	-26254000	6.53E+18	-7.65E+21	1
Dihydrolevoglucosenone	0.11	-66.85	-144418000	0.00E+00	0.00E+00	2
Dimethyl isosorbide	0.22	-237.21	-170102000	0.00E+00	0.00E+00	2

Table S3: Molar volume coefficient for probe solvents

Probe solvent	A	B	C	D	E	Ref
Dimethyl sulfoxide	1.110	0.250	729.000	0.330	0	1
Dimethylformamide	0.890	0.230	650.600	0.290	0	1
□-valerolactone	0.990	0.280	727.000	0.350	0	1
Methanol	2.330	0.270	512.500	0.250	0	1
Benzonitrile	0.722	0.246	702.300	0.288	0	1
Acetone	1.193	0.254	508.100	0.292	0	1
Dimethyl carbonate	1.037	0.258	557.000	0.296	0	1
Toluene	0.879	0.271	591.750	0.292	0	1
o-xylene	0.715	0.265	630.300	0.274	0	1
□-pinene	0.623	0.283	644.000	0.297	0	1
Ethyl acetate	0.900	0.259	523.300	0.278	0	1
Tetrahydrofuran	1.254	0.281	540.150	0.291	0	1
Benzene	1.054	0.270	562.050	0.290	0	1
D-limonene	0.597	0.280	653.000	0.293	0	1
Ethylbenzene	0.708	0.263	617.260	0.284	0	1
Butyl acetate	0.678	0.264	575.400	0.293	0	1
Ethanol	1.629	0.275	514.000	0.232	0	1
1-Propanol	1.246	0.273	536.800	0.240	0	1
Nonane	0.463	0.254	594.600	0.286	0	1
Decane	0.411	0.252	617.700	0.286	0	1
Undecane	0.367	0.249	639.000	0.286	0	1
Dodecane	0.333	0.247	658.000	0.286	0	1
Hexadecane	0.224	0.233	722.000	0.268	0	1
1-Pentanol	0.818	0.267	588.100	0.253	0	1
p-xylene	0.689	0.261	616.200	0.279	0	1
Methyl acetate	1.114	0.258	506.500	0.271	0	1
Propyl acetate	0.730	0.255	549.730	0.277	0	1
Cyclohexane	0.890	0.274	553.800	0.286	0	1
Cyclohexene	0.930	0.271	560.400	0.289	0	1
Dihydrolevoglucosenone	2.993	0.499	705.279	0.661	0	1
Dimethyl isosorbide	1.414	0.419	604.405	0.362	0	1

Table S4: Extended Antoine coefficients for probe solvents

Probe solvent	A	B	C	D	E	Ref
Dimethyl sulfoxide	56.273	-7620.600	-4.628	4.38E-07	2	1
Dimethylformamide	78.832	-7968.690	-8.061	1.42E-10	3.5	1
□-valerolactone	83.640	-8784.300	-8.888	4.53E-06	2	1
Methanol	82.718	-6904.500	-8.862	7.47E-06	2	1
Benzonitrile	55.040	-7363.830	-4.506	1.95E-18	6	1
Acetone	57.947	-5355.270	-5.211	1.24E-14	5	1
Dimethyl carbonate	86.268	-7151.070	-9.406	8.25E-09	3	1
Toluene	76.945	-6729.800	-8.179	5.30E-06	2	1
o-xylene	60.616	-6814.800	-5.435	5.94E-18	6	1
□-pinene	97.752	-8491.600	-11.124	5.33E-06	2	1
Ethyl acetate	66.824	-6227.600	-6.410	1.79E-17	6	1
Tetrahydrofuran	54.898	-5305.400	-4.763	1.43E-17	6	1
Benzene	119.299	-7326.390	-15.853	1.69E-02	1	1
D-limonene	75.574	-8079.700	-7.560	8.39E-18	6	1
Ethylbenzene	76.626	-7330.300	-7.900	4.72E-09	3	1
Butyl acetate	122.820	-9253.200	-14.990	1.05E-05	2	1
Ethanol	73.304	-7122.300	-7.142	2.89E-06	2	1
1-Propanol	84.660	-8307.240	-8.580	7.51E-18	6	1
Nonane	109.350	-9030.400	-12.882	7.85E-06	2	1
Decane	112.730	-9749.600	-13.245	7.13E-06	2	1
Undecane	131.000	-11143.000	-15.855	8.19E-06	2	1
Dodecane	137.470	-11976.000	-16.698	8.09E-06	2	1
Hexadecane	162.644	-15734.400	-19.620	6.43E-09	3	1
1-Pentanol	114.748	-10643.300	-12.858	1.25E-17	6	1
p-xylene	59.356	-6631.000	-5.274	6.52E-18	6	1
Methyl acetate	73.467	-6096.940	-7.518	1.14E-11	4	1
Propyl acetate	115.160	-8433.900	-13.934	1.03E-05	2	1
Cyclohexane	51.087	-5226.400	-4.228	9.76E-18	6	1
Cyclohexene	88.184	-6624.900	-10.059	8.26E-06	2	1
Dihydrolevoglucosenone	52.643	-8186.340	-3.965	2.59E-07	0	1
Dimethyl isosorbide	87.451	-10787.170	-8.776	0	0	3

Table S5: Fitted parameters for the Van't Hoff model

Probe solvent	a	b	R^2	AARD %
Nonane	738.42	2.12	0.87	1.84
Decane	425.73	2.92	0.89	0.95
Undecane	290.13	3.33	0.90	0.61
Dodecane	98.97	3.85	0.78	0.34
Hexadecane	315.48	3.54	0.85	0.86
Methanol	-799.76	0.96	0.67	3.69
Ethanol	872.72	-2.47	0.83	2.60
1-Propanol	3872.01	-8.60	1.00	1.40
1-Pentanol	5137.99	-10.17	1.00	0.08
o-xylene	1954.82	-1.78	0.85	5.18
p-xylene	2809.76	-3.93	0.93	4.88
Dimethylformamide	432.18	-2.70	0.81	1.35
□-valerolactone	5525.05	-12.56	1.00	0.42
Benzene	473.59	0.44	1.00	0.17
D-limonene	290.73	2.73	0.74	1.12
□-pinene	439.40	2.49	0.88	1.05
Benzonitrile	5355.61	-11.94	1.00	0.71
Toluene	3697.09	-6.49	1.00	0.27
Tetrahydrofuran	211.67	0.18	1.00	0.09
Acetone	2605.68	-6.07	0.99	1.99
Methyl acetate	2958.57	-6.49	1.00	0.65
Ethyl acetate	172.60	0.59	0.88	0.41
Propyl acetate	227.78	1.37	0.82	0.69
Butyl acetate	3483.51	-5.57	0.98	2.92
Cyclohexane	1908.53	-1.52	0.98	1.70
Cyclohexene	2320.62	-2.39	0.95	3.35
Dimethyl sulfoxide	2690.23	-8.79	0.99	1.28
Ethylbenzene	2896.59	-4.09	0.97	3.21
Dimethyl carbonate	1596.41	-3.28	0.83	4.59
Dimethyl isosorbide	5449.62	-10.29	0.99	3.56
Dihydrolevoglucosenone	6155.00	-12.43	1.00	0.85

Table S6: Hansen solubility parameters for probe solvents at different column temperatures

Temperature	408.15 K				423.15 K				438.15 K			
Probe solvent	$\square_{t}$	$\square_{D}$	$\square_{P}$	$\square_{H}$	$\square_{t}$	$\square_{D}$	$\square_{P}$	$\square_{H}$	$\square_{t}$	$\square_{D}$	$\square_{P}$	$\square_{H}$
Nonane	12.96	12.96	0.00	0.00	12.56	12.56	0.00	0.00	12.15	12.15	0.00	0.00
Decane	13.29	13.29	0.00	0.00	12.92	12.92	0.00	0.00	12.54	12.54	0.00	0.00
Undecane	13.50	13.50	0.00	0.00	13.15	13.15	0.00	0.00	12.79	12.79	0.00	0.00
Dodecane	13.70	13.70	0.00	0.00	13.36	13.36	0.00	0.00	13.02	13.02	0.00	0.00
Hexadecane	14.31	14.31	0.00	0.00	14.02	14.02	0.00	0.00	13.73	13.73	0.00	0.00
Methanol	25.00	11.67	10.34	19.54	24.35	11.16	10.00	19.19	23.68	10.61	9.63	18.85
Ethanol	22.53	12.75	7.49	17.00	21.93	12.23	7.26	16.70	21.31	11.67	7.01	16.40
1-Propanol	20.97	13.15	5.87	15.25	20.44	12.68	5.71	14.98	19.89	12.19	5.54	14.71
1-Pentanol	18.57	13.45	3.97	12.18	18.14	13.07	3.89	11.96	17.70	12.68	3.80	11.75
o-xylene	15.51	15.24	0.89	2.72	15.12	14.86	0.87	2.67	14.73	14.47	0.86	2.62
p-xylene	15.17	14.90	0.88	2.72	14.77	14.50	0.86	2.67	14.35	14.09	0.85	2.62
Dimethylformamide	21.44	14.70	12.07	9.90	20.96	14.31	11.83	9.73	20.47	13.91	11.58	9.55
□-valerolactone	19.39	14.71	10.63	6.84	19.02	14.40	10.46	6.71	18.65	14.08	10.29	6.59
Benzene	15.12	15.02	0.00	1.75	14.61	14.50	0.00	1.72	14.07	13.96	0.00	1.69
D-limonene	15.42	14.86	1.61	3.77	15.07	14.52	1.59	3.70	14.71	14.17	1.56	3.64
□-pinene	14.24	14.11	0.89	1.75	13.91	13.77	0.88	1.72	13.56	13.43	0.86	1.69
Benzonitrile	17.41	15.14	8.11	2.89	17.06	14.81	7.98	2.84	16.70	14.48	7.84	2.79
Toluene	15.16	15.01	1.22	1.75	14.71	14.56	1.19	1.72	14.24	14.10	1.17	1.69
Tetrahydrofuran	15.99	13.52	4.84	7.01	15.45	13.01	4.71	6.89	14.90	12.47	4.56	6.76
Acetone	15.74	11.77	8.46	6.13	15.07	11.17	8.13	6.02	14.36	10.52	7.78	5.92
Methyl acetate	14.89	11.93	5.92	6.66	14.28	11.34	5.70	6.54	13.62	10.70	5.45	6.42
Ethyl acetate	14.59	12.40	4.42	6.31	14.04	11.85	4.27	6.20	13.45	11.27	4.11	6.09
Propyl acetate	14.48	12.33	3.66	6.66	14.01	11.86	3.55	6.54	13.51	11.38	3.44	6.42

Butyl acetate	14.45	12.97	3.19	5.52	14.01	12.54	3.11	5.42	13.56	12.09	3.03	5.33
Cyclohexane	13.68	13.68	0.00	0.18	13.19	13.19	0.00	0.17	12.69	12.69	0.00	0.17
Cyclohexene	14.20	14.03	1.29	1.75	13.71	13.54	1.25	1.72	13.20	13.03	1.22	1.69
Dimethyl sulfoxide	23.46	15.93	14.72	8.94	23.01	15.58	14.48	8.78	22.56	15.23	14.23	8.62
Ethylbenzene	15.12	15.06	0.53	1.23	14.71	14.66	0.52	1.20	14.30	14.24	0.51	1.18
Dimethyl carbonate	15.45	12.47	3.31	8.50	14.98	12.01	3.22	8.35	14.49	11.53	3.12	8.20
Dimethyl isosorbide	18.03	15.51	6.75	6.23	17.68	15.19	6.69	6.07	17.32	14.87	6.64	5.91
Dihydrolevoglucosenone	20.43	16.78	10.13	5.77	20.13	16.51	10.06	5.62	19.84	16.23	10.00	5.48

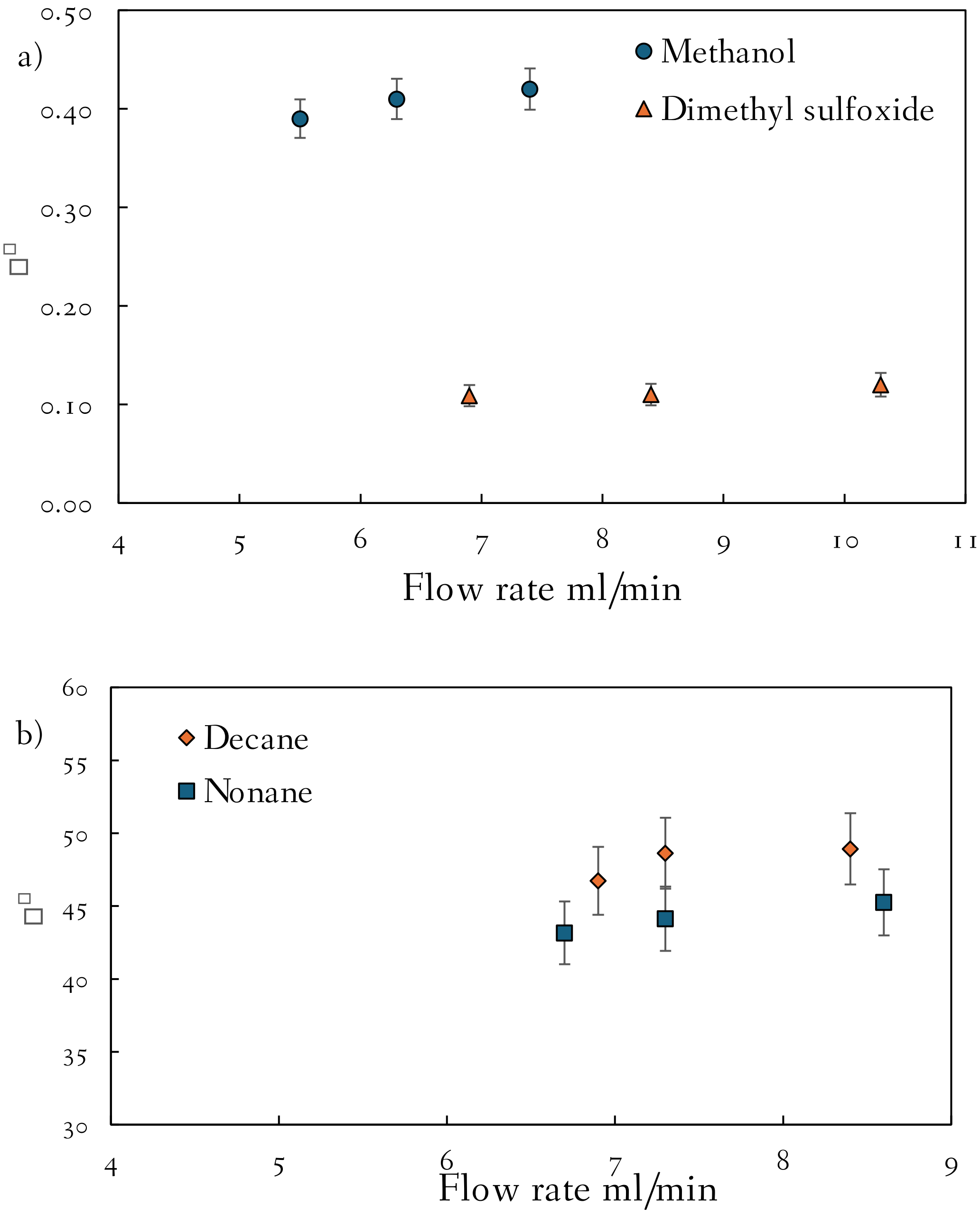

Figure S3: □[□] as a function of flow rate at 438.15 K column temperature (a) Methanol and Dimethyl sulfoxide (b) Decane and Nonane

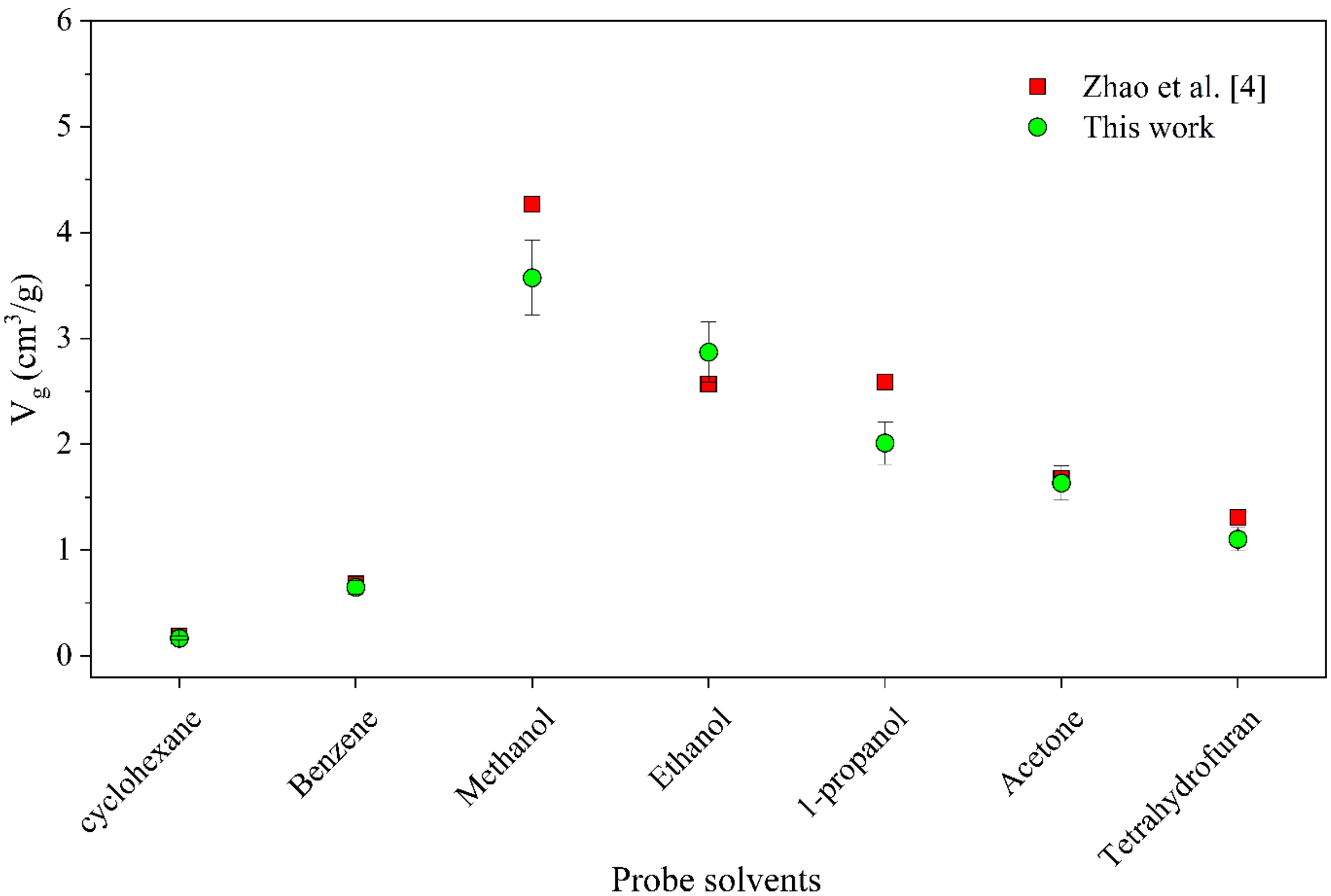

Figure S4: EHL-specific retention volume (V_g) for probe solvents at 423.15 K

Table S7: Flory-Huggins Interaction Parameter for probe solvents in EHL

Probe solvent	408.15 K	423.15 K	438.15 K
Nonane	4.56	4.52	4.39
Decane	4.53	4.49	4.41
Undecane	4.54	4.51	4.45
Dodecane	4.52	4.50	4.47
Hexadecane	4.50	4.48	4.42
Methanol	1.13	1.08	1.19
Ethanol	1.44	1.27	1.23
1-Propanol	2.41	2.02	1.70
1-Pentanol	3.58	3.12	2.68
o-xylene	4.04	3.96	3.67
p-xylene	3.96	3.80	3.44
Dimethylformamide	-0.13	-0.16	-0.24
δ-valerolactone	2.27	1.76	1.31
Benzene	2.93	2.86	2.80
D-limonene	4.21	4.20	4.13
α-pinene	4.36	4.33	4.25
Benzonitrile	2.42	1.92	1.49
Toluene	3.74	3.39	3.08
Tetrahydrofuran	2.11	2.06	2.01
Acetone	1.79	1.48	1.27
Methyl acetate	2.16	1.85	1.59
Ethyl acetate	2.22	2.18	2.12
Propyl acetate	2.99	2.95	2.89
Butyl acetate	3.88	3.62	3.25
Cyclohexane	4.30	4.07	3.93
Cyclohexene	4.52	4.22	4.08
Dimethyl sulfoxide	-0.33	-0.51	-0.82
Ethylbenzene	4.02	3.82	3.49
Dimethyl carbonate	1.97	1.91	1.64
Dimethyl isosorbide	3.90	3.49	2.95
Dihydrolevoglucosenone	3.90	3.37	2.84

Table S8: Energy of adsorption for the probe solvents on the surface of EHL

Probe solvent	-∆E^A (kJ/mol)	R^2	AARD %
Nonane	-31.98	0.9930	2.09
Decane	-38.78	0.9984	1.20
Undecane	-43.95	0.9993	0.88
Dodecane	-49.61	0.9997	0.62
Hexadecane	-64.26	0.9993	1.28
Methanol	-39.23	0.987	3.46
Ethanol	-27.80	0.9883	2.33
1-Propanol	-4.79	0.9173	1.11
1-Pentanol	-0.63	0.7855	0.26
o-xylene	-20.68	0.8976	5.47
p-xylene	-12.51	0.7833	5.14
Dimethylformamide	-35.85	0.9969	1.56
□-valerolactone	-1.00	0.8863	0.28
Benzene	-24.02	1.0000	0.02
D-limonene	-38.30	0.9980	1.33
□-pinene	-33.29	0.9976	1.28
Benzonitrile	-0.23	0.0907	0.57
Toluene	-1.22	0.9823	0.13
Tetrahydrofuran	-24.76	1.0000	0.05
Acetone	-3.76	0.7173	1.82
Methyl acetate	-0.54	0.4524	0.46
Ethyl acetate	-26.69	0.9991	0.62
Propyl acetate	-29.71	0.9985	0.90
Butyl acetate	-6.32	0.7055	3.18
Cyclohexane	-11.58	0.9710	1.54
Cyclohexene	-8.32	0.8035	3.17
Dimethyl sulfoxide	-72.09	0.9977	2.69
Ethylbenzene	-11.35	0.8689	3.43
Dimethyl carbonate	-17.33	0.8871	4.83
Dimethyl isosorbide	-13.15	0.8749	3.88
Dihydrolevoglucosenone	-1.65	0.8048	0.62

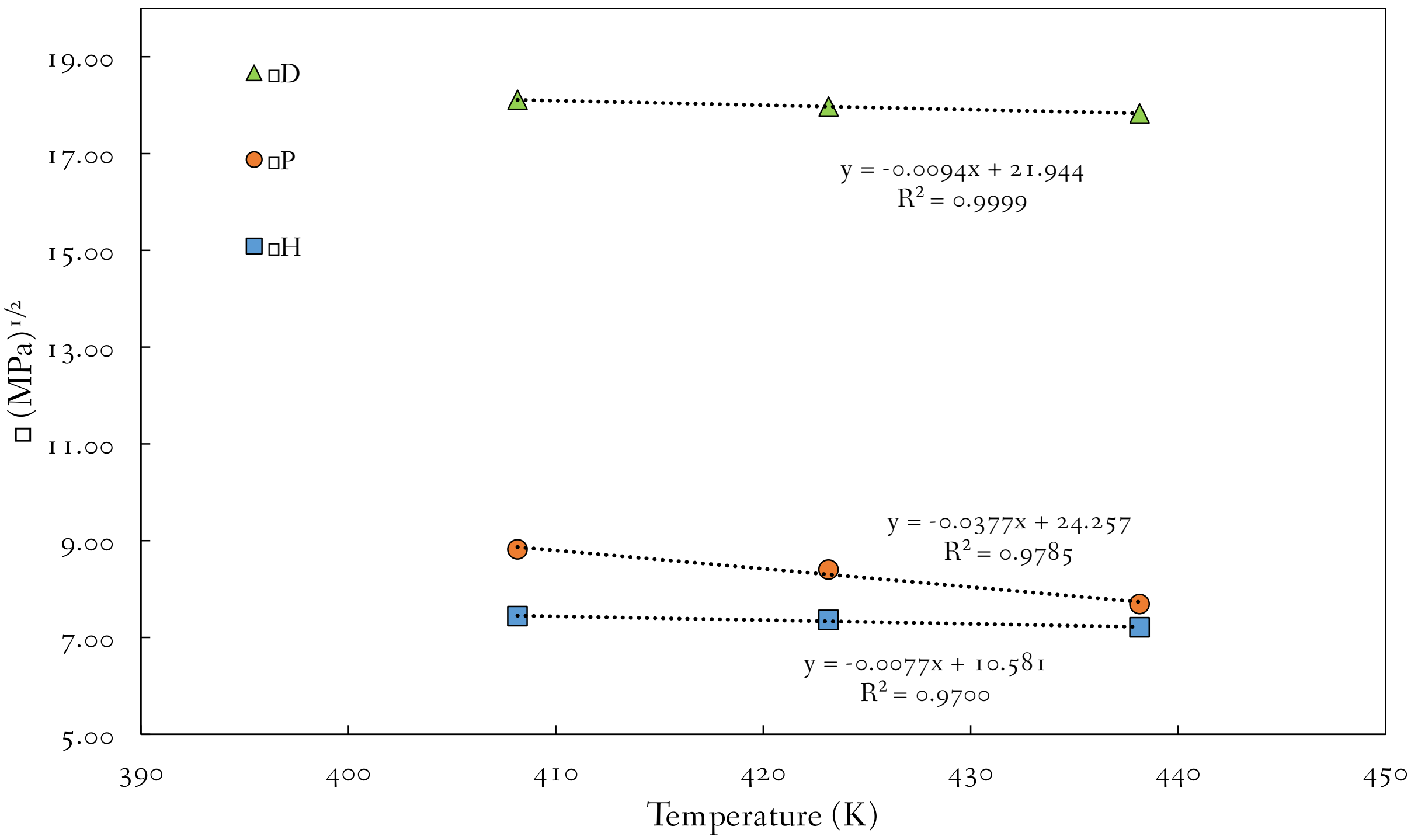

Figure S5: Relationship between Hansen solubility parameter and temperature for EHL

Table S9: EHL dissolved (wt. %) of EHL in different pure solvents via gravimetric method[a] at 298.15 K

Solvent	$m_{initial}$ (g)	$m_{dissolved}$ (g)	EHL dissolved (wt. %)[b]
Nonane	1.996	0.048	2.41 (± 0.7)
Dodecane	2.005	0.029	1.45 (± 0.8)
1-Propanol	2.024	0.524	25.87 (± 1.5)
1-Pentanol	2.024	0.203	10.04 (± 1.2)
o-xylene	2.005	0.078	3.88 (± 1.1)
p-xylene	2.008	0.100	4.98 (± 0.8)
Dimethylformamide	2.102	2.033	96.73 (± 1.2)
□-valerolactone	2.031	1.563	77.93 (± 1.9)
Benzene	2.016	0.278	13.78 (± 1.3)
□-pinene	2.026	0.093	4.60 (± 1.1)
Toluene	2.036	0.156	7.67 (± 0.7)
Tetrahydrofuran	2.008	1.453	71.34 (± 1.1)
Acetone	2.018	1.518	73.20 (± 0.9)
Methyl acetate	1.999	0.583	36.51 (± 1.5)
Ethyl acetate	2.015	0.471	28.40 (± 1.6)
Propyl acetate	1.995	0.190	15.10 (± 1.4)
Butyl acetate	2.034	0.142	8.50 (± 1.1)
Cyclohexane	2.006	0.047	2.34 (± 0.9)
Cyclohexene	2.003	0.044	2.22 (± 0.6)
Dimethyl sulfoxide	2.030	2.013	99.14 (± 1.3)
Ethylbenzene	2.000	0.067	3.33 (± 0.7)
Dimethyl carbonate	2.026	0.448	22.11 (± 1.4)

[a] The standard uncertainty is $u(m) = 0.002$ g and $u(T) = 0.5$ K

[b] EHL dissolved (wt.%) = $\frac{m_{dissolved}}{m_{initial}} * 100$

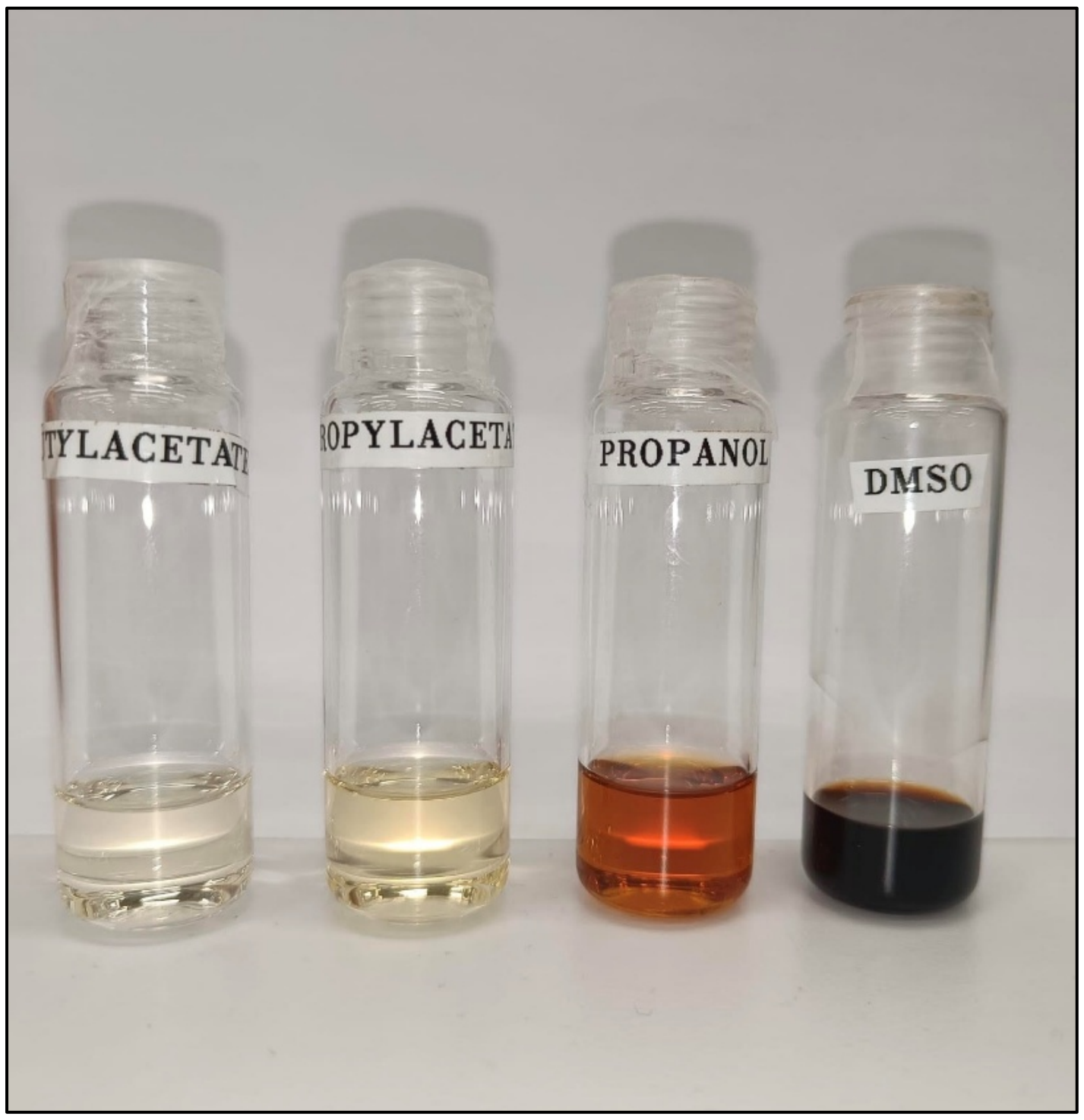

Figure S6: Visual representation of dissolved EHL in solvent. Samples are taken from a syringe coupled with a 0.45 μm pore size polypropylene filter. Left to right: Butyl acetate, Propyl acetate, 1-Propanol, and Dimethyl sulfoxide

Table S10: Solubility threshold criteria for determination of interaction radius

Solubility threshold criteria (% EHL dissolved)	*R*₀	Solvent in sphere	Solvent outside sphere
80	4.60	3	28
75	6.20	4	27
60[a]	8.98	8	23

[a] *R*₀ for the 60% and 70% solubility thresholds is the same since no solvent has a % EHL dissolved value between 60–70% solubility thresholds in Table S9

Table S11: Relative energy difference values and solvent classification for the 31 solvents in EHL at 298.15 K with a solubility threshold of 70%

Probe solvent	Score	RED	Solvent classification
Nonane	0	1.89	Poor
Decane	0	1.87	Poor
Undecane	0	1.86	Poor
Dodecane	0	1.85	Poor
Hexadecane	0	1.83	Poor
Methanol	0	1.84	Poor
Ethanol	0	1.51	Poor
1-Propanol	0	1.41	Poor
1-Pentanol	0	1.34	Poor
o-xylene	0	1.49	Poor
p-xylene	0	1.50	Poor
Dimethylformamide	1	0.51	Good
▱-valerolactone	1	0.51	Good
Benzene	0	1.62	Poor
D-limonene	0	1.39	Poor
▱-pinene	0	1.63	Poor
Benzonitrile	1	0.81	Good
Toluene	0	1.49	Poor
Tetrahydrofuran	1	0.96	Good
Acetone	1	0.87	Good
Methyl acetate	0	1.04	Poor
Ethyl acetate	0	1.14	Poor
Propyl acetate	0	1.29	Poor
Butyl acetate	0	1.29	Poor
Cyclohexane	0	1.78	Poor
Cyclohexene	0	1.04	Poor
Dimethyl sulfoxide	1	0.46	Good
Ethylbenzene	0	1.61	Poor
Dimethyl carbonate	0	1.30	Poor
Dimethyl isosorbide	1	0.74	Good
Dihydrolevoglucosenone	1	0.32	Good

Reference

(1) Design Institute for Physical Property Data (DIPPR Project 801). AIChE, 2024. https://app.knovel.com/hotlink/toc/id:kpDIPPRPF7/dippr-project-801-full/dippr-project-801-full
(2) Tsonopoulos, C. An empirical correlation of second virial coefficients. AIChE Journal 1974, 20 (2), 263–272. DOI: https://doi.org/10.1002/aic.690200209
(3) Durand, M. Propriétés physico-chimiques, fonctionnelles et applicatives des éthers courts d'isosorbide. 2010. https://api.semanticscholar.org/CorpusID:92437843.
(4) Zhao, G.; Liu, X.; Ren, S.; Tan, W.; Fang, G. Quantitative comparison of surface properties of enzymatic hydrolysis lignin before and after degradation. Industrial Crops and Products 2018, 125, 468-472. DOI: https://doi.org/10.1016/j.indcrop.2018.09.020.
